# The role of WRKY transcription factors in exogenous potassium (K^+^) response to NaCl stress in *Tamarix ramosissima*

**DOI:** 10.3389/fgene.2023.1274288

**Published:** 2023-11-20

**Authors:** Yahui Chen, Xuanyi Zhang, Yunlong Fan, Dezong Sui, Jiang Jiang, Lei Wang

**Affiliations:** ^1^ Jiangsu Academy of Forestry, Nanjing, China; ^2^ Collaborative Innovation Center of Sustainable Forestry in Southern China of Jiangsu Province, Nanjing Forestry University, Nanjing, China; ^3^ Faculty of Science Department of Statistics, University of British Columbia, Vancouver, BC, Canada

**Keywords:** exogenous potassium (K^+^), halophyte, NaCl stress, transcriptome, WRKY transcription factor

## Abstract

**Introduction:** Soil salinization poses a significant challenge to plant growth and vitality. Plants like *Tamarix ramosissima* Ledeb (*T. ramosissima*), which are halophytes, are often integrated into planting schemes tailored for saline environments. Yet, the role of WRKY transcription factors in *T. ramosissima*, especially under sodium chloride (NaCl) stress mitigated by exogenous K^+^ application, is not well-understood. This research endeavors to bridge this knowledge gap.

**Methods:** Using Pfam protein domain prediction and physicochemical property analysis, we delved into the WRKY genes in *T. ramosissima* roots that are implicated in counteracting NaCl stress when aided by exogenous K^+^ applications. By observing shifts in the expression levels of WRKY genes annotated to the KEGG pathway under NaCl stress at 0, 48, and 168 h, we aimed to identify potential key WRKY genes.

**Results:** We found that the expression of 56 WRKY genes in *T. ramosissima* roots responded to exogenous K^+^ application during NaCl stress at the indicated time points. Particularly, the expression levels of these genes were primarily upregulated within 168 h. From these, 10 WRKY genes were found to be relevant in the KEGG pathways. Moreover, six genes, namely *Unigene0024962*, *Unigene0024963*, *Unigene0010090*, *Unigene0007135*, *Unigene0070215*, and *Unigene0077293*, were annotated to the Plant-pathogen interaction pathway or the MAPK signaling pathway in plants. These genes exhibited dynamic expression regulation at 48 h with the application of exogenous K^+^ under NaCl stress.

**Discussion:** Our research highlights that WRKY transcription factors can modulate the activation or inhibition of related genes during NaCl stress with the application of exogenous K^+^. This regulation enhances the plant’s adaptability to saline environments and mitigates the damage induced by NaCl. These findings provide valuable gene resources for future salt-tolerant *Tamarix* breeding and expand our understanding of the molecular mechanisms of WRKY transcription factors in alleviating NaCl toxicity.

## 1 Introduction

Soil salinization poses a significant environmental issue worldwide. Reports indicate that approximately 8.31 × 106 km^2^ of the earth’s soil is affected by salinity (Li et al., 2014), and projections warn that due to escalating soil salinity, as much as 50% of the world’s arable land could be lost by mid-century. This exacerbation is predominantly caused by environmental degradation, global warming, and inappropriate irrigation practices (Ludwig et al., 2018). High salt levels are known to inhibit plant growth severely, a phenomenon first highlighted in Boyer’s 1984 study (Boyer, 1982). The ramifications on global agriculture are considerable, with an estimated economic toll exceeding 27 billion US dollars annually due to loss in productivity (Qadir et al., 2014). Therefore, devising strategies to utilize saline-alkaline land efficiently and promoting the development and cultivation of salt-resistant crops is of paramount importance (Savary et al., 2020).

Salt stress, the detrimental impact of highly saline soils on plant growth, affects many stages of a plant’s life, from seed germination to flowering and fruiting (Park et al., 2013). This often results in reduced germination rates, slower growth, decreased plant height, and leaf wilting (Hao et al., 2021). High Na^+^ concentrations in saline-alkali soils cause osmotic stress, obstructing the absorption of water and nutrients, leading to a nutrient-water imbalance (Mauro et al., 2022). Potassium (K^+^) is a key cation in plant cells, improving plant resilience to both abiotic stresses, such as salt, drought, and heavy metals, and biotic stresses like fungi (Leigh and Jones, 1984; Amtmann et al., 2008). High salt content in soil hinders K^+^ absorption due to competition with Na^+^, leading to a decrease in the K^+^/Na^+^ ratio, which results in excessive Na^+^ in plants, inhibiting growth and potentially causing plant death (Shabala and Cuin, 2008; Wang et al., 2019). Plant enrichment with K^+^ can enhance their water status, biomass, and salt tolerance (Tittal et al., 2021; Johnson et al., 2022). Under salt stress, the Na^+^ content in plants greatly exceeds K^+^ levels (Chakraborty et al., 2016). To prevent Na^+^ toxicity, plants must maintain a high K^+^/Na^+^ ratio to regulate the Na^+^/K^+^ balance (Chakraborty et al., 2016). Thus, boosting K^+^ absorption and minimizing Na^+^ accumulation in plants is a prime strategy against salt stress (Munns and Tester, 2008; Shabala and Cuin, 2008; Chen et al., 2022a).

Transcription factors (TFs) are also known as trans-acting factors and are specialized proteins that can regulate plant growth and development. Transcription factors can bind to specific DNA sequences (cis-acting elements) in the promoter region of target genes through their DNA-binding domains (DBD). This interaction allows them to control the specific expression of target genes in different tissues, cells, or under various environmental conditions in plants (Singh et al., 2002). Transcription factor families such as WRKY, V-myb avian myeloblastosis viral oncogene homolog (MYB), NAM, ATAF1/2 and CUC2 (NAC), Basic leucine zipper (bZIP), APETALA2/ethylene responsive factor (AP2/ERF), and Basic helix-loop-helix (bHLH) play pivotal regulatory and molecular switch roles in the intricate salt stress signaling network. By activating or suppressing the expression of specific genes or a set of genes, these transcription factors ensure their targeted expression. The products resulting from this gene expression further control the expression of downstream genes or directly act to protect plants from the damage induced by salt stress (Khan et al., 2018). It’s worth noting that WRKY transcription factors have been recognized over the past two decades as instrumental in regulating plant responses to biotic and abiotic stresses (Schluttenhofer and Yuan, 2015).

WRKY transcription factors are a plant-specific transcription factor family, which is one of the largest transcription factor families in higher plants (Ulker and Somssich, 2004; Cheng et al., 2021). These factors regulate gene transcription by identifying and binding to the W-box, a conserved DNA binding site. As a result, they play roles in various plant functions, including growth, development, defense against pathogens, responses to both biological and environmental stress, hormone signal processing, secondary metabolism, and other processes (Li and Zhou, 2014; Wei et al., 2022). WRKY transcription factors can also regulate the expression of related genes by binding cis-elements or interacting with other regulatory factors to play an important role in plant growth and development and resist abiotic stress (Wei et al., 2022). In particular, WRKY transcription factors can positively or negatively regulate the expression of related genes when plants are subjected to salt stress, thus making plants tolerant to salt. Dai et al. reported that *Fortunella crassifolia FcWRKY40* can directly activate the expression of serine/threonine protein kinase gene *FcSOS2* in SOS pathway, indirectly regulate the expression of *FcSOS1* and *FcSOS3* genes, promote Na^+^ effection, and positive response to salt stress (Dai et al., 2018). Research by Song and colleagues revealed that *SbWRKY50*, found in Sorghum, had a negative impact on the salt response by down-regulating the expression of *AtSOS1*, a Na^+^/H^+^ reverse transporter gene in *Arabidopsis* (Song et al., 2020). The overexpression of *OsWRKY40* in rice decreased the tolerance of rice to high salt (Tao et al., 2011). *GhWRKY25* is overexpressed and its tolerance to salt stress is enhanced, while in transgenic tobacco, it has relatively weak tolerance to drought stress (Liu et al., 2016).

As halophytes, *Tamarix* plants utilize their innate physiological and metabolic mechanisms to enhance their resistance to saline conditions (Gao et al., 2011; Gupta et al., 2014; Che et al., 2019; Bahramsoltani et al., 2020). Plants of the *Tamarix* plants have remarkable resilience to high salt environments, making them essential for restoring arid, desert, and saline regions (Yang et al., 2021; Sun et al., 2022). Recognizing their value, China has significantly invested in Tamarix cultivation as part of its ecological conservation initiatives. Additionally, *Tamarix* plants play a vital role in Chinese herbal medicine due to their medicinal properties (Duan et al., 2022). Studies by Flowers and Colmer indicate that halophytes can successfully complete their life cycle even when subjected to NaCl concentrations exceeding 200 mM (Flowers and Colmer, 2008; Liu et al., 2022). Typically, *Tamarix ramosissima* Ledeb (*T*. *ramosissima*), a representative of the *Tamarix* plants, is used as a restoration species in saline-alkali areas (Duan et al., 2022). *T*. *ramosissima* can sustain growth under NaCl stress up to concentrations of 100 mM, beyond which the plant’s growth is negatively impacted. Interestingly, NaCl concentrations exceeding 200 mM do not result in the plant’s death but significantly reduce its growth rate (Chen et al., 2023). However, halophytes like *T*. *ramosissima* have the ability to increase K^+^ retention when under salt stress, facilitating their adaptation to a range of saline and alkaline conditions (Garthwaite et al., 2005). Studies have discovered that the roots of *T*. *ramosissima* can resist NaCl stress using 10 mM KCl and can continue to grow even under 200 mM NaCl stress (Chen et al., 2022b; Chen et al., 2022c). In this manuscript, *T*. *ramosissima* was used as the research subject. We studied and analyzed the role of *WRKY* genes in *T*. *ramosissima* in response to NaCl stress when exogenous K^+^ is applied. By delving into the transcriptome, we identified key candidate *WRKY* genes and their essential metabolic pathways. This provides a scientific theoretical foundation for the selection of salt-tolerant varieties and the involvement of WRKY transcription factors in alleviating the toxic effects of NaCl.

## 2 Materials and methods

Throughout this study, root samples of *T. ramosissima* under the stress of 200 mM NaCl with the application of exogenous K^+^ at 0, 48, and 168 h were collected for transcriptome sequencing. The data obtained underwent processing, including the screening of Differential expressed genes (DEGs) and database annotation, then identifing the *WRKY*-related genes. Using Pfam_Scan, protein domains of these genes were predicted (Finn et al., 2016). Protparam (https://web.expasy.org/protparam/) provided predictions on the basic physicochemical properties of the WRKY gene family members (Gasteiger et al., 2003), while CELLOv.2.5 (http://cello.life.nctu.edu.tw/) was employed for their subcellular localization. Subsequent analysis focused on the KEGG pathways annotated by the identified *WRKY* genes. A phylogenetic tree of the key *WRKY* candidate genes was then constructed. Lastly, the accuracy of the transcriptome data for these genes was validated using Quantitative Real-Time PCR (qRT-PCR) (Supplementary Figure S1).

### 2.1 Plant materials and treatment

The *T. ramosissima* with similar growth (seedling age: 5 months) were selected. They were cultivated in a 24-well hydroponic box with 1/2 Hoagland nutrient solution (replaced every 3 days), and the experimental treatments were applied 2 months later. The group with 1/2 Hoagland nutrient solution was set as the control group. The seedlings cultivated in 200 mM NaCl 1/2 Hoagland nutrient solution and 200 mM NaCl + 10 mM KCl 1/2 Hoagland nutrient solution were the treatment groups, with 8 plants per group (repeated 3 times). The nutrient solution was replaced every 3 days, with 24 seedlings for all treatments. At the same time, root samples were collected at 0, 48, and 168 h after treatment and stored in a −80°C freezer for transcriptome sequencing.

### 2.2 Transcriptome sequencing and differentially expressed gene screening

The collected *T. ramosissima* root samples were entrusted to biotechnology company (Gene Denovo, Guangzhou, China) for transcriptome sequencing. During the experiment, ultrasound was used to break down the mRNA. (Note: mRNA is Oligo (dT) magnetic beads enriched to eukaryotic mRNA with a polyA tail). The mRNA (fragmentation) is then used as the basis to synthesize the double strand of cDNA. The purified double-stranded cDNA was screened out using about 200bp and PCR amplification was performed. Finally, the database was constructed using PCR products, which were purified by AMPure XP beads Chen et al., 2022b). The raw data obtained (*WRKY* genes in the roots of *T*. *ramosissima* - NCBI Short Reads Archive database: SRP356215) was analyzed. DEGs were identified based on the criteria of FDR < 0.05 (corrected *p*-value) and |log_2_FC| > 1. The DEGs were annotated to the Gene Ontology (GO) (Conesa et al., 2005) and Kyoto Encyclopedia of Genes and Genomes (KEGG) (Michael et al., 2000) databases.

### 2.3 Prediction of Pfam protein structural domains

Through transcriptome sequencing results, members of the WRKY family annotated in the NCBI database were obtained from the roots of the *T. ramosissima*. Then, the obtained WRKY proteins were matched in the Pfam database using the Pfam_Scan program, yielding protein structure-related annotation information for the *WRKY* genes (Finn et al., 2016), and the final members of the WRKY gene family were obtained. The basic physicochemical properties of the members of the WRKY gene family were analyzed and predicted online using Protparam (https://web.expasy.org/protparam/), including Molecular weight, Theoretical pI, Grand average of hydropathicity (GRAVY), Instability index, and Aliphatic index, etc. Finally, the obtained WRKY proteins were subjected to subcellular localization prediction analysis using the online website CELLOv.2.5 (http://cello.life.nctu.edu.tw/).

### 2.4 Quantitative Real-Time PCR (qRT-PCR) verification

Seven *WRKY* candidate genes were randomly chosen to validate the transcriptome sequencing accuracy. Total RNA from *T*. *ramosissima* root samples was extracted using the RNAprep pure kit (Tiangeng, Beijing, China), and cDNA was synthesized with the Prime Script™ DEG II 1st Strand cDNA Synthesis Kit (Takara, Beijing, China). Primers were designed for the above *WRKY* genes (Supplementary Table S1). However, after diluting the cDNA template 8 times, qRT-PCR experiments were conducted according to the instructions of the TB Green Premix ExTaq (Tli RNaseH Plus) kit (Takara, RR420A) by Takara company. The reaction system was set to 20 μL, including 10 μL TB Green Premix Ex Taq, 0.8 μL upstream primer (10 μmol L^−1^), 0.8 μL downstream primer (10 μmol L^−1^), 1 μL DNA template, and 7.4 μL dd H_2_O. Three replicates were set for each sample, with all operations performed on ice. Amplification of each sample was executed using the ABI ViiA™ 7 Real-time PCR system (ABI, California, United States). The PCR amplification protocol included an initial denaturation at 95°C for 30 s, followed by 40 cycles of denaturation at 95°C for 5 s and annealing at 60°C for 30 s. The melting curve process involved heating at 95°C for 5 s, then 60°C for 1 min, gradually increasing to 95°C, and finally cooling to 50°C for 30 s (Yang et al., 2022). Each gene was biologically replicated 3 times, with *Tubulin* as the internal reference gene. The 2^−ΔΔCT^ method was used to calculate the relative expression levels (Livak and Schmittgen, 2001).

### 2.5 Experiment data processing

Data computations such as mean and standard deviations were performed using Microsoft Excel (Microsoft, Washington, United States). The prediction of Pfam protein structural domains was made through the Pfam_Scan program. The construction of graphs was facilitated by the Origin 2019 software (OriginLab, Massachusetts, United States). Phylogenetic trees were developed utilizing the MEGA 11 software (MEGA software, Pennsylvania, United States). Lastly, LSD analysis was executed using the ANOVA tool in the SPSS 26.0 software (SPSS, New York, United States).

## 3 Results

### 3.1 Prediction analysis of Pfam protein structure domains

The transcript data results revealed 61 *WRKY*-related genes. Subsequent predictions of these 61 WRKY gene protein sequences in the Pfam database (Supplementary Table S3) showed that 5 *WRKY* genes (*Unigene0009588*, *Unigene0016969*, *Unigene0023528*, *Unigene0052366*, and *Unigene0062497*) were not present in the Pfam database The results show that the start-to-end positions of the structure domains of the Unigene-encoded protein sequences predicted by the HMM models of these 56 *WRKY* genes are 1-489, the HMM length is all 21-59, the bit score is between 23.7-96.4, the HMM acc is PF03106.15, the clan is CL0274, and the PfamA_definition is WRKY DNA-binding domain. Finally, there are 56 *WRKY* genes present in the roots of the *T. ramosissima* under the exogenous NaCl stress.

### 3.2 Identification and physicochemical property analysis of WRKY genes in the roots of *T. ramosissima*

**FIGURE 1:**
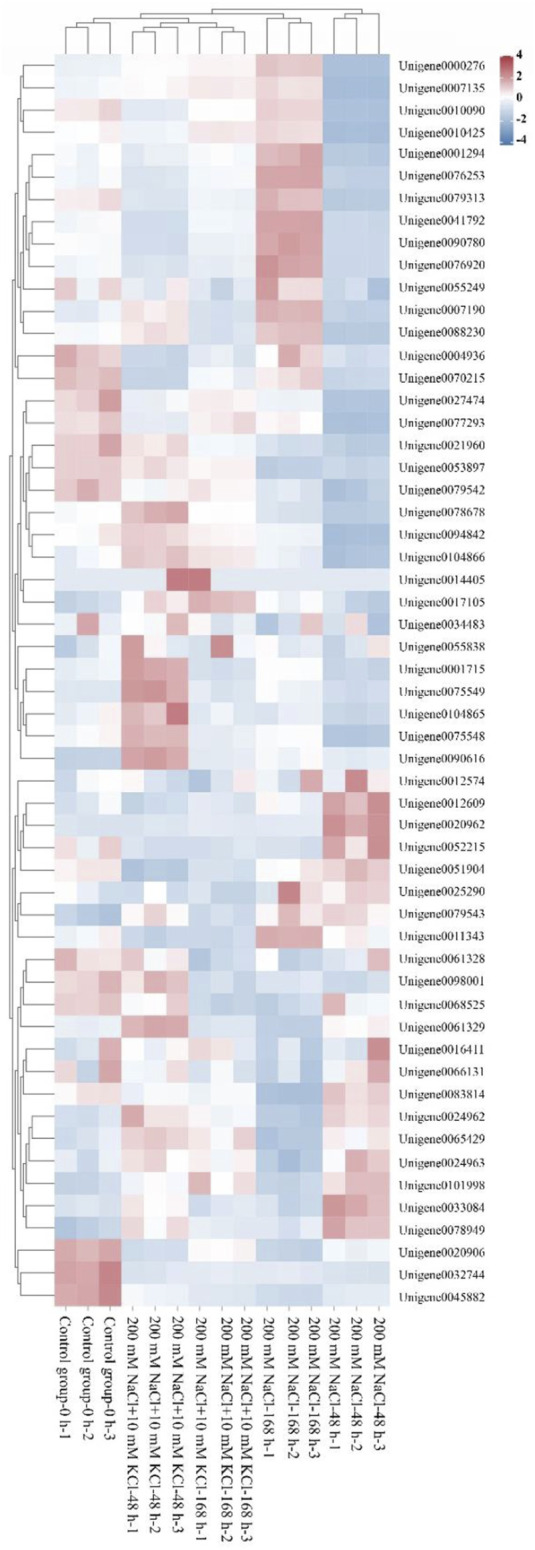
Clustering heatmap of the *WRKY* candidate genes (A heatmap clustering analysis was performed on the 56 identified *WRKY* candidate genes across various samples).

The Protparam online tool was used to analyze the physicochemical properties of the 56 *WRKY* genes, ande the 56 *WRKY* genes were analyzed by clustering heat map with each sample (Figure 1). The results show (Table 1) that the number of amino acids ranges from 39 to 728 aa, indicating a large variation in the number of amino acids; the molecular weight ranges from 4385.91 (*Unigene0075549*) to 69860.14 (*Unigene0032744*) Da, which is proportional to the amino acid content; Theoretical pI ranges from 4.70 (*Unigene0077293*) to 10.26 (*Unigene0041792*), among which 27 members are basic proteins (pI > 7), and 29 members are acidic proteins (pI < 7); only 4 WRKY proteins have an Instability index less than 40, indicating that most of them are unstable proteins. The results of hydrophilicity/hydrophobicity of the WRKY proteins obtained are all less than 0, indicating that these 56 WRKY proteins are hydrophilic. The subcellular prediction results show that all WRKY proteins are located in the nuclear.

**TABLE 1:** WRKY gene family information of *T*. *ramosissima*

Gene ID	ORF/aa	Molecular weight	Theoretical pI	GRAVY	Instability index	Aliphatic index	Subcellular localization
*Unigene0076253*	187	21108.72	5.56	−0.921	59.01	27.70	Nuclear
*Unigene0061328*	51	5689.37	8.62	−0.749	23.26	36.47	Nuclear
*Unigene0001294*	122	13952.30	9.30	−1.339	56.76	38.36	Nuclear
*Unigene0041792*	106	11842.45	10.26	−1.024	43.40	38.68	Nuclear
*Unigene0070215*	114	13023.43	10.19	−1.255	38.85	40.26	Nuclear
*Unigene0004936*	160	18114.62	5.50	−1.116	38.13	41.31	Nuclear
*Unigene0078949*	249	28240.02	6.58	−1.168	68.74	45.78	Nuclear
*Unigene0012609*	232	25655.25	8.76	−0.919	51.71	45.78	Nuclear
*Unigene0077293*	180	19497.00	4.70	−0.872	46.13	46.72	Nuclear
*Unigene0052215*	609	67186.50	8.85	−0.863	56.14	48.21	Nuclear
*Unigene0021960*	355	39213.13	6.40	−0.895	59.03	49.30	Nuclear
*Unigene0010090*	632	68852.13	6.51	−0.918	53.66	49.56	Nuclear
*Unigene0007135*	575	62627.58	6.26	−0.774	55.26	49.70	Nuclear
*Unigene0020962*	252	28622.79	6.77	−0.981	44.02	49.96	Nuclear
*Unigene0011343*	329	35264.87	6.97	−0.775	62.94	50.46	Nuclear
*Unigene0079542*	501	53725.20	5.36	−0.854	52.09	50.48	Nuclear
*Unigene0007190*	255	27861.47	5.92	−0.968	59.44	50.63	Nuclear
*Unigene0090616*	130	15194.16	9.64	−1.072	41.92	50.92	Nuclear
*Unigene0025290*	195	22255.82	9.32	−1.010	50.31	51.90	Nuclear
*Unigene0098001*	566	60735.69	8.46	−0.836	62.84	52.99	Nuclear
*Unigene0075548*	305	33070.37	5.65	−0.678	57.39	53.18	Nuclear
*Unigene0104865*	494	53656.32	8.11	−0.845	51.73	53.56	Nuclear
*Unigene0032744*	665	69860.14	6.23	−0.589	53.48	55.85	Nuclear
*Unigene0065429*	615	65432.66	5.95	−0.672	49.31	55.97	Nuclear
*Unigene0079543*	513	56066.51	5.96	−0.743	47.55	56.24	Nuclear
*Unigene0079313*	550	58821.01	6.22	−0.598	46.49	56.80	Nuclear
*Unigene0068525*	203	21788.06	9.33	−0.741	48.30	56.80	Nuclear
*Unigene0034483*	189	21744.23	6.35	−0.953	42.14	57.20	Nuclear
*Unigene0075549*	39	4385.91	9.24	−0.810	49.69	57.44	Nuclear
*Unigene0017105*	583	63000.11	6.96	−0.698	49.66	58.04	Nuclear
*Unigene0012574*	282	32207.34	8.32	−0.883	51.76	58.40	Nuclear
*Unigene0104866*	506	55331.41	6.93	−0.788	64.38	58.42	Nuclear
*Unigene0083814*	361	38413.24	9.59	−0.650	45.70	58.67	Nuclear
*Unigene0016411*	560	61016.73	6.98	−0.832	48.87	60.73	Nuclear
*Unigene0033084*	375	40205.78	9.17	−0.716	61.11	61.15	Nuclear
*Unigene0024963*	232	26361.25	8.90	−1.047	33.15	61.25	Nuclear
*Unigene0020906*	244	27466.33	8.21	−0.981	55.39	61.97	Nuclear
*Unigene0061329*	370	40649.28	10.02	−0.661	56.21	61.97	Nuclear
*Unigene0024962*	496	54325.27	6.38	−0.900	40.26	62.26	Nuclear
*Unigene0014405*	140	16207.29	9.92	−0.878	53.83	62.71	Nuclear
*Unigene0101998*	345	38036.14	5.64	−0.806	49.49	62.75	Nuclear
*Unigene0053897*	364	40888.56	8.63	−0.830	51.81	62.75	Nuclear
*Unigene0045882*	581	61880.46	8.22	−0.569	50.31	62.93	Nuclear
*Unigene0078678*	340	38231.54	6.83	−0.816	45.20	63.94	Nuclear
*Unigene0076920*	404	43452.25	6.33	−0.475	44.34	64.73	Nuclear
*Unigene0055838*	280	31828.55	8.21	−0.814	63.52	65.89	Nuclear
*Unigene0094842*	392	42493.39	5.71	−0.491	48.35	66.71	Nuclear
*Unigene0055249*	508	54877.26	6.46	−0.623	54.71	67.28	Nuclear
*Unigene0010425*	152	16724.90	6.73	−0.630	48.43	67.30	Nuclear
*Unigene0066131*	134	15205.10	9.36	−0.794	60.77	67.61	Nuclear
*Unigene0000276*	331	36214.41	6.63	−0.663	48.43	67.79	Nuclear
*Unigene0027474*	390	42967.37	9.71	−0.724	55.91	68.26	Nuclear
*Unigene0088230*	343	38413.54	6.12	−0.762	47.12	69.39	Nuclear
*Unigene0051904*	334	36844.91	9.87	−0.645	55.36	71.23	Nuclear
*Unigene0090780*	175	18904.46	9.40	−0.421	49.35	71.49	Nuclear
*Unigene0001715*	564	61473.25	7.79	−0.378	47.66	79.95	Nuclear

Note: ORF/aa means Number of amino acids, GRAVY means Grand average of hydropathicity.

### 3.3 Changes in the expression levels of WRKY genes

According to our focus on the expression levels of 56 *WRKY* genes (Supplementary Figure S2), five genes (U*nigene0066131*, *Unigene0061329*, *Unigene0053897*, *Unigene0025290*, and *Unigene0101998*) showed an initial upregulation followed by downregulation after 48 and 168 h of NaCl stress. However, their expression levels were first downregulated and then upregulated after 48 and 168 h of exogenous K^+^ application under NaCl stress. Another ten genes (*Unigene0024962*, *Unigene00249623*, *Unigene0079543*, *Unigene0104866*, *Unigene0094842*, *Unigene0078949*, *Unigene0078678*, *Unigene0075549*, *Unigene0065429*, and *Unigene0033084*) showed initial upregulation followed by downregulation under NaCl stress at 48 and 168 h. However, they consistently exhibited an upregulated trend in their expression levels after 48 and 168 h of exogenous K^+^ application under NaCl stress. Notably, *Unigene0090616* showed an upregulated trend in expression levels, whether it was under NaCl stress at 48 and 168 h or under NaCl stress with exogenous K^+^ application at 48 and 168 h.

### 3.4 WRKY genes annotated to KEGG pathway

Ten DEGs of the 56 WRKY genes found in the roots of *T*. *ramosissima* were annotated in the related KEGG pathway (Supplementary Table S2) when exogenous K^+^ was applied at 0, 48 and 168 h under NaCl stress. 6 *WRKY* genes (Unigene0007135, *Unigene0010090*, *Unigene0014405*, *Unigene0052215*, *Unigene0070215* and *Unigene0077293*) were annotated in the plant-interaction pathway (ko04626) and the MAPK signaling pathway-plant pathway (ko04016), The 6 *WRKY* genes were classified into Organismal Systems, and the Environmental Information Processing in KEGG A. KEGG B classified Environmental adaptation and Signal transduction. *Unigene0024962*, *Unigene0024963*, *Unigene0079542* and *Unigene0079543* are annotated for the Plant-pathogen interaction pathway (ko04626). In addition, these 4 *WRKY* genes were classified into Organismal Systems in KEGG A and Environmental adaptation in KEGG B. According to the expression levels of the 10 *WRKY* genes at 0, 48 and 168 h under NaCl stress (Supplementary Figure S2), The expression levels of 3 *WRKY* genes (*Unigene0024962*, *Unigene0024963*, and *Unigene0079543*) increased at 48 h and 168 h when applying exogenous K^+^ under NaCl stress. Notably, the expression levels of *Unigene0024962* and *Unigene0024963* consistently rose at both 48 and 168 h with exogenous K^+^ under NaCl stress. However, under NaCl stress alone, their expression levels first increased at 48 h and then decreased at 168 h.

### 3.5 Analysis of plant-pathogen interaction pathway

As inferred from the analysis of the Plant-pathogen interaction pathway (Table 2), In the 200 mM NaCl-48 h vs. 200 mM NaCl + 10 mM KCl-48 h (N-48 h vs. N + K-48 h) comparison group, 99 DEGs were significantly enriched into the Plant-pathogen interaction pathway (*p* < 0.05). Among them, 65 DEGs were upregulated, while 34 were downregulated. The 7 *WRKY* genes (*Unigene0052215*, *Unigene0010090*, *Unigene0007135*, *Unigene0070215 Unigene0077293*, *Unigene0024962* and *Unigene00249623*) have been annotated for Plant-pathogen interaction pathway in N-48 h vs. N + K-48 h comparison group, and the expression level changed (Supplementary Figure S3). In addition, *Unigene0052215, Unigene0024962,* and *Unigene00249623* were down-regulated, while *Unigene0010090*, *Unigene0007135, Unigene0070215,* and *Unigene0077293* were upregulated, suggesting their roles in NaCl stress resistance. In the comparison of the 200 mM NaCl-168 h and 200 mM NaCl + 10 mM KCl-168 h groups, 74 DEGs were identified in the Plant-pathogen interaction pathway. Of these, 21 DEGs were upregulated and 53 were downregulated. Notably, 3 *WRKY* genes (*Unigene0070215, Unigene0024962*, and *Unigene00249623*) were associated with this pathway. The expression level of *Unigene0070215* was downregulated, while the expression levels of *Unigene0024962* and *Unigene00249623* were upregulated.

**TABLE 2:** Analysis of Plant-pathogen interaction pathway.

Pathway	Gene numbers	Class	*p*-Value	Up	Down
N-48 h vs. N + K-48 h					
Plant–pathogen interaction	99	Organismal Systems	0.013055	65	34
N-168 h vs. N + K-168 h					
Plant–pathogen interaction	74	Organismal Systems	0.186311	21	53

Note: N-48 h: 200 mM NaCl-48 h, N + K-48 h:200 mM NaCl + 10 mM KCl-48 h, N-168 h: 200 mM NaCl-168 h, N + K-168 h: 200 mM NaCl + 10 mM KCl-168 h.

### 3.6 Analysis of MAPK signaling pathway-plant pathway

Based on the MAPK signaling pathway-plant pathway (Table 3), in the 200 mM NaCl-48 h vs. 200 mM NaCl + 10 mM KCl-48 h comparison group, 57 DEGs were enriched to MAPK signaling Pathway-plant pathway. Among them, 37 DEGs showed upregulated expression levels, while 20 DEGs exhibited downregulated expression. *Unigene0052215*, *Unigene0010090*, *Unigene0007135*, *Unigene0070215* and *Unigene0077293* belong to *WRKY* genes that have been annotated to MAPK signaling the N-48 h vs. N + K-48 h comparison group Pathway-plant pathway, and the expression level was changed (Supplementary Figure S4). Among them, in addition to the downregulated expression of *Unigene0052215*, the upregulated expression of *Unigene0010090*, *Unigene0007135*, *Unigene0070215* and *Unigene0077293* actively participate in the resistance to NaCl stress. In the N-168 h vs. N + K-168 h comparison group, 54 DEGs were significantly enriched to MAPK signaling Pathway-plant pathway (*p* < 0.05). Among them, 19 DEGs showed upregulated expression levels, while 35 DEGs exhibited downregulated expression. Only *Unigene0070215* belongs to the *WRKY* gene and has been annotated to MAPK signaling pathway-plant in N-168 h vs. N + K-168 h comparison group pathway, and its expression level was still downregulated.

**TABLE 3:** Analysis of MAPK signaling pathway-plant pathway.

Pathway	Gene numbers	Class	*p*-Value	Up	Down
N-48 h vs. N + K-48 h					
MAPK signaling pathway-plant pathway	57	Environmental Information Processing	0.297932	37	20
N-168 h vs. N + K-168 h					
MAPK signaling pathway-plant pathway	54	Environmental Information Processing	0.067500	19	35

Note: N-48 h: 200 mM NaCl-48 h, N + K-48 h:200 mM NaCl + 10 mM KCl-48 h, N-168 h: 200 mM NaCl-168 h, N + K-168 h: 200 mM NaCl + 10 mM KCl-168 h.

### 3.7 Candidate key WRKY genes were constructed for phylogenetic tree analysis

*Unigene0024962* and *Unigene00249623*, which belong to the WRKY gene family (WRKY transcription factor 1), have both been annotated in the Plant-pathogen interaction pathway. Under NaCl stress, their expression levels initially increase at 48 h and then decrease at 168 h. However, after exogenous K^+^ application under NaCl stress, their expression levels continue to show an increasing trend at both 48 and 168 h. Thus, it can be inferred that *Unigene0024962* and *Unigene00249623* are key candidate genes in the WRKY family. The protein amino acid sequences of Unigene0024962 and Unigene0024963 were compared on the NCBI using Blast, resulting in 15 homologous species were selected (Supplementary Table S4). A phylogenetic tree constructed with MEGA software revealed that Unigene0024962 shares a close phylogenetic relationship with *Tamarix hispida* (Supplementary Figure S5).

### 3.8 Quantitative Real-Time PCR (qRT-PCR) validation of candidate WRKY genes

Seven *WRKY* candidate genes were selected for qRT-PCR validation to verify the accuracy of our transcriptome sequencing outcomes. The patterns observed in qRT-PCR expression closely matched those from the transcriptomic study (Supplementary Figure S6). This affirms the integrity and dependability of the transcriptome data acquired. Hence, this research lays a solid foundation for identifying *WRKY* genes in *T*. *ramosissima* roots that enhance salinity resilience and mitigate the adverse impacts of NaCl stress.

## 4 Discussion

*Tamarix* plants has developed a complex regulatory network to adapt to abiotic stress (Zhu, 2016). Transcription factors are the most important regulatory factors in all abiotic stress responses (Yao et al., 2016). The WRKY family is large, *Arabidopsis thaliana* had 74 *WRKY* genes (Eulgem et al., 2000), cotton had 116 *WRKY* genes (Dou et al., 2014), rice (*Oryza sativa*) had 102 *WRKY* genes (Ross et al., 2007), *Solanum Lycopersicum* had 81 *WRKY* genes (Huang et al., 2012), carrot (*Daucus carota* L.) had 95 *WRKY* genes (Li et al., 2016), and Castor Bean (*Ricinus communis* L.) had 58 *WRKY* genes (Zou et al., 2016). In this study, 56 *WRKY* genes were found in the roots of *T. ramosissima* under NaCl stress at 48 h and 168 h. Further analysis of these 56 *WRKY* genes can help to understand the response mechanism of *T. ramosissima* to NaCl stress under exogenous K^+^ application. To identify salt tolerance genes and related metabolic pathways to provide a scientific and theoretical basis for further research on salt tolerance in *T. ramosissima*.

WRKY transcription factors are essential in plant responses to biotic and abiotic stresses (Jiang et al., 2017). Particularly, it plays a crucial role in responding to salt stress in various plants (Huang et al., 2022). A WRKY transcription factor *PbWRKY40* from *Pyrus betulaefolia* functions positively in salt tolerance and modulating organic acid accumulation by regulating *PbVHA-B1* expression (Lin et al., 2022). *Ahwrky75* was overexpressed in the M34 mutant of “salt-tolerant” peanut, which improved the salt tolerance of transgenic peanut (Zhu et al., 2021). In transgenic tobacco, overexpression of the WRKY transcription factor gene of Begonia (*MbWRKY5*) can improve drought tolerance and salt tolerance (Han et al., 2018). in *Solanum lycopersicum*, *SlWRKY8* plants overexpressed showed tolerance to salt stress (Gao et al., 2020). In this study, 37 DEGs were upregulated, and 24 DEGs were downregulated in the N-48 h vs. N + K-48 h comparison group. In the N-168 h vs. N + K-168 h comparison group, 33 DEGs were upregulated, and 28 DEGs were downregulated. Additionally, overexpression of the *WRKY* gene *DgWRKY1* or *DgWRKY3* in chrysanthemum plants increased their salt tolerance by *dendranthema grandiflorum* (Liu et al., 2013). However, K^+^ can better regulate Na^+^ uptake under salt conditions, which plays a vital role in regulating plant physiological processes to enable plants to survive under stress conditions, especially in improving their tolerance to salt stress (Çolpan et al., 2013). Furthermore, the introduction of K^+^ helped mitigate the negative impacts of Na^+^, enhanced the uptake of K^+^, and raised the K^+^/Na^+^ ratio under conditions of NaCl stress (Carden et al., 2003). It is worth noting that halophytes have a solid competitive advantage in maintaining K^+^ stability under a high NaCl stress (Ardie et al., 2009). In this study, expression level at 48 and 168 h increased first and then decreased under NaCl stress. However, the expression level of the candidate key *WRKY* genes (*Unigene0024962* and *Unigene0024963*) were upregulated after the application of exogenous K^+^ for 48 and 168 h under NaCl stress. Therefore, the increased expression level of *Unigene0024962* and *Unigene0024963* may be influenced by the addition of exogenous K^+^. In brief, the results showed that *WRKY* genes expression in the roots of *T. ramosissima* was consistently upregulated within 168 h after applying exogenous NaCl stress, which improved the tolerance and resistance of *T. ramosissima* to NaCl stress.

Under conditions of salt stress in grapes (*Vitis vinifera*), *VvWRKY28* has the ability to boost the accumulation levels of enzymatic antioxidants (superoxide dismutase, peroxidase, and catalase) as well as proline. These changes counteract the over-accumulation of reactive oxygen species and support osmotic balance in cells, thus increasing the plant’s overall salt tolerance (Liu et al., 2022). In *T*. *ramosissima*, when exposed to 200 mM NaCl stress for 48 h and 168h, the leaves had more activity of certain helpful enzymes. At the same time, there was more proline in the roots. These changes helped the seeds handle salt better. (Chen et al., 2022b; Chen et al., 2022c). In this study, the expression level of the WRKY gene (Unigene0*020906*) was upregulated after applying exogenous K^+^ for 48 and 168 h under 200 mM NaCl stress. Therefore, it may play an essential role in increasing the main antioxidant activities and proline content accumulation of *T. ramosissima*. Besides, abiotic stresses such as high temperature, salinity and drought affect plant growth. They also affect plant defense response to pathogens through enhancement or inhibition of pathogens, to regulate plant response to pathogen infection (Pandey and Senthil-Kumar, 2019). In particular, one of the earliest signaling events after a plant sense an invading pathogen is the activation of mitogen activated protein kinase (MAPK) signaling cascades. They are able to correlate the perception of external stimuli with cellular responses, and MAPK signaling networks play specific and overlapping roles in controlling the activity of a large number of transcription factors, enzymes, hormones, peptides, and antibacterial chemicals, among others (Taj et al., 2014). WRKY transcription factors have been shown to regulate plant defense responses (Rushton and Somssich, 1998). WRKY transcription factors regulate plant defense responses (Rushton and Somssich, 1998). Their activity is modulated by phosphorylation through mitogen-activated protein kinase (Ishihama and Yoshioka, 2012; Adachi et al., 2015). WRKY protein is involved in *Arabidopsis thaliana* response to bacterial flagellin flg22 as a transcriptional activator at the end of the PAMP signaling cascade. Flagellin flg22 in *Arabidopsis thaliana* initiates the entire MEKK1-MKK4/5-MAPK3/6 MAPK cascade pathway, leading to the activation of WRKY22/29 expressions. These expressions function after the flagellin receptor FLS2, a leucine-rich repeat (LRR) receptor kinase, and further stimulate MAPK cascades, enhancing plant resistance (Sheen et al., 2002). Simultaneously, AtMKK1 and AtMKK2 in *Arabidopsis Thaliana* are also associated with biological and abiotic stress responses as part of a signaling cascade that includes MEKK1 and MPK4 (Teige et al., 2004). Nevertheless, AtMKK2 is also involved in mediating salt signaling (Hadiarto et al., 2006). It is worth noting that Mkk1 is a positive regulator of flg22 activation of MPK3, MPK4, MPK6, and a negative regulator of flg22 activation of gene expression, and its role is very complex (Meszaros et al., 2006). MPK3 and MPK6 are two mitogen-activated protein kinases (MAPK or MPK) that play key roles in plant resistance by regulating various defense responses (Meng et al., 2013). *AtWRKY33* mutants in *Arabidopsis Thaliana* are very sensitive to NaCl, and overexpression of *AtWRKY33* can enhance tolerance to NaCl stress (Jiang and Deyholos, 2009; Li et al., 2011). In this study, FLS2 activated downstream mitogen-activated protein kinase (MEKK) in both the Plant-pathogen interaction pathway and the MAPK signaling Pathway-plant pathway, MKK1/MKK2 → MPK4 or MKK4/MKK5 → MPK3/MPK6 (+p is the process of protein phosphorylation). They transmit these signals into the nucleus to transcription factors (WRKY 22/WRKY29 or WRKY25/WRKY33) that activate the transcription of genes associated with immune defense. The expression levels of *Unigene0010090*, *Unigene0007135*, *Unigene0070215* and *Unigene0077293* were upregulated in the N-48 h vs. N + K-48 h comparison group. In the comparison group of N-168 h vs. N + K-168 h, the expression levels of *Unigene0024962* and *Unigene0024963* were upregulated. Therefore, it is concluded that these genes actively participate in the resistance to NaCl stress when exogenous K^+^ is applied for 48 h under NaCl stress. However, with the increase of K^+^ addition time, the damage of NaCl stress was alleviated, *T. ramosissima* could maintain normal growth, and the genes involved in regulation were reduced.

To conclude, the results of this manuscript suggest that the result of this manuscript suggest that the WRKY transcription factors are involved in *T*. *ramosissima*‘s response to external K^+^ under NaCl stress. A significant number of *WRKY*-related genes are upregulated and take part in essential metabolic pathways, thereby creating a comprehensive defense mechanism to help *T*. *ramosissima* resist NaCl stress. Notably, two *WRKY* candidate genes, *Unigene0024962* and *Unigene00249623*, play pivotal roles within these pathways. They are identified as key *WRKY* genes worthy of further investigation.

## 5 Conclusion

The WRKY transcription factor family is one of the largest transcriptional regulatory families in higher plants, involved in regulating plant growth and development, and responding to biotic and abiotic stresses. They play an indispensable role in the normal life activities of plants, being involved in various aspects of the plant’s lifecycle. In this study, the roots of *T*. *ramosissima*, under exogenous potassium in response to salt stress at 48 h and 168 h, saw the involvement of the WRKY transcription factor family in the growth and development processes of *T*. *ramosissima*, as well as the construction of its defense system. A significant number of *WRKY* genes actively upregulated their expression levels to resist NaCl stress through the plant-pathogen interaction pathway and the MAPK signaling pathway-plant pathway, alleviating the damage caused by NaCl and thus maintaining the normal growth of *T*. *ramosissima*. Notably, two key candidate *WRKY* genes (*Unigene0024962* and *Unigene00249623*) actively upregulated their expression levels in the plant-pathogen interaction pathway after exogenous potassium application under NaCl stress, playing a crucial role in the *T*. *ramosissima* root system’s mitigation of NaCl stress. They can serve as key salt tolerance genes in the *Tamarix* plants for further exploration.

## Data Availability

The datasets presented in this study can be found in online repositories. The names of the repository/repositories and accession number(s) can be found below: https://www.ncbi.nlm.nih.gov/, SRP356215.
