# Supplementary material for: The role of WRKY transcription factors in exogenous potassium (K+) response to NaCl stress in Tamarix ramosissima
Source: Front Genet. 2023 Nov 20;14:1274288. doi: 10.3389/fgene.2023.1274288 (PMC10694239; doi:10.3389/fgene.2023.1274288)
Supplement: Supplementary file 1 [file DataSheet1.PDF]

Supplementary Table S1. Sequences of specific primers

Primer name	Primer sequence (5'-3')
Unigene0007135	F: CCACCGCTATTACTACGACCTCCA<br>R: TCCATCCTCTGATTGCTTGCTTGTC
Unigene0010090	F: ACACTACTACAACACCACCACCAAT<br>R: ACTCCAGGAATGCGTCGTCTCT
Unigene0077293	F: CCACGACCTCTACCACCTCTACTTC<br>R: GACTGCTGTTGTTAGTTGCTGGAGA
Unigene0024962	F: CTGCGGCTGTTGATACGGTTATTCA<br>R: TCTGGCATCACACCAACTTCCTCT
Unigene0024963	F: TCTTCAACACGGGCATCCAACATT<br>R: ACAGCACATACAGTCTCAACCACAA
Unigene0079542	F: CATAACCATCCCAAGCCTCCACAAA<br>R: CAAGAGCTGCACCACCACATCC
Unigene0079543	F: GCAAGGTAGAGCAAGTAGAGCGATT<br>R: GCCAGCGATAGCCATCATAAAGGAT
Tubulin	F: GCTGAGATTACAACCGCTG<br>R: CTGTTCGTTTGGTCTTGATT

Note: F means Forward primer, R means Reversed primer.

Supplementary Table S2. *WRKY* genes in the roots of *T. ramosissima*

Gene ID	Description	KEGG_A_class	KEGG_B_class	KEGG Pathway	Log_2 fold-change	
					N-48 h vs. N + K-48 h	N-168 h vs. N + K-168 h
Unigene0000276	Probable WRKY transcription factor 40	-	-	-	3.75	−0.54
Unigene0001294	Probable WRKY transcription factor 12 isoform X2	-	-	-	2.10	−1.06
Unigene0001715	Probable WRKY transcription factor 31	-	-	-	0.31	0.63
Unigene0004936	Probable WRKY transcription factor 43 isoform X2	-	-	-	0.44	−2.04
Unigene0007135	WRKY33-1_1	Organismal Systems; Environmental Information Processing	Environmental adaptation; Signal transduction	ko04626;ko04016	3.52	−0.22
Unigene0007190	WRKY transcription factor 9	-	-	-	1.30	−0.45
Unigene0009588	WRKY transcription factor 50	-	-	-	−0.41	0.17
Unigene0010090	Transcription factor WRKY33	Organismal Systems; Environmental Information Processing	Environmental adaptation; Signal transduction	ko04626;ko04016	2.29	−0.80
Unigene0010425	WRKY transcription factor 7	-	-	-	4.58	−0.59
Unigene0011343	Predicted: probable WRKY transcription factor 71	-	-	-	−0.86	−1.56
Unigene0012574	WRKY transcription factor 22	-	-	-	−1.05	−0.53
Unigene0014405	Predicted: WRKY transcription factor 22	Organismal Systems;Environmental Information Processing	Environmental adaptation;Signal transduction	ko04626;ko04016	5.44	5.44
Unigene0012609	Probable WRKY transcription factor 57	-	-	-	−0.62	−0.36

Unigene0016411	WRKY transcription factor 10	-	-	-	0.01	0.30
Unigene0016969	WRKY transcription factor 50	-	-	-	-0.47	-0.48
Unigene0017105	Probable WRKY transcription factor 20 isoform X2	-	-	-	0.39	0.09
Unigene0020906	WRKY transcription factor 28	-	-	-	0.45	1.31
Unigene0020962	DNA-binding WRKY	-	-	-	-3.36	-1.93
Unigene0021960	WRKY transcription factor 6	-	-	-	1.23	0.97
Unigene0023528	WRKY transcription factor WRKY24-like	-	-	-	-0.67	1.15
Unigene0024962	WRKY transcription factor 1	Organismal Systems	Environmental adaptation	ko04626	-0.12	0.40
Unigene0024963	WRKY transcription factor 1	Organismal Systems	Environmental adaptation	ko04626	-0.16	0.72
Unigene0025290	Predicted: probable WRKY transcription factor 75	-	-	-	-3.31	-1.55
Unigene0027474	WRKY transcription factor 4	-	-	-	1.04	-0.01
Unigene0032744	WRKY DNA-binding protein 72	-	-	-	0.20	-0.10
Unigene0033084	WRKY transcription factor	-	-	-	-0.61	0.45
Unigene0034483	Predicted: probable WRKY transcription factor 43	-	-	-	0.14	0.34
Unigene0041792	Predicted: probable WRKY transcription factor 48	-	-	-	2.01	-2.67
Unigene0045882	WRKY transcription factor 72B-like isoform X1	-	-	-	-0.11	0.80
Unigene0051904	WRKY transcription factor 2	-	-	-	-0.64	-0.81
Unigene0052215	WRKY33-2_1	Organismal Systems;Environmental Information Processing	Environmental adaptation;Signal transduction	ko04626;ko04016	-3.96	-0.78
Unigene0052366	WRKY transcription factor	-	-	-	-0.47	0.21

Unigene0053897	Predicted: probable WRKY transcription factor 40 isoform X1	-	-	-	1.51	2.04
Unigene0055249	WRKY transcription factor 12	-	-	-	0.19	−0.33
Unigene0055838	Probable WRKY transcription factor 53	-	-	-	0.81	0.69
Unigene0061328	Predicted: probable WRKY transcription factor 74	-	-	-	−1.17	0.97
Unigene0061329	Predicted: probable WRKY transcription factor 74	-	-	-	−0.59	1.85
Unigene0062497	WRKY transcription factor 19	-	-	-	0.54	−0.84
Unigene0065429	Probable WRKY transcription factor 14	-	-	-	0.05	0.81
Unigene0066131	WRKY transcription factor 6	-	-	-	−0.27	0.35
Unigene0068525	WRKY transcription factor	-	-	-	−1.59	1.54
Unigene0070215	Probable WRKY transcription factor 27, partial	Organismal Systems;Environmental Information Processing	Environmental adaptation;Signal transduction	ko04626;ko04016	3.10	−6.00
Unigene0075548	Predicted: probable WRKY transcription factor 69 isoform X1	-	-	-	1.53	0.62
Unigene0075549	Probable WRKY transcription factor 65	-	-	-	2.02	1.59
Unigene0076253	WRKY transcription factor 7	-	-	-	2.59	−1.82
Unigene0076920	WRKY transcription factor 55	-	-	-	6.01	−2.91
Unigene0077293	WRKY transcription factor	Organismal Systems;Environmental Information Processing	Environmental adaptation;Signal transduction	ko04626;ko04016	2.07	−0.42
Unigene0078678	WRKY transcription factor 9	-	-	-	2.93	1.64
Unigene0078949	WRKY transcription factor 23−like	-	-	-	−0.47	0.34

Unigene0079313	Probable WRKY transcription factor 31	-	-	-	1.24	−1.05
Unigene0079542	WRKY transcription factor 11	Organismal Systems	Environmental adaptation	ko04626	0.77	0.24
Unigene0079543	WRKY transcription factor 11	Organismal Systems	Environmental adaptation	ko04626	−0.46	−0.05
Unigene0083814	WRKY transcription factor	-	-	-	−0.41	1.20
Unigene0088230	WRKY transcription factor 9	-	-	-	1.98	−0.28
Unigene0090616	WRKY transcription factor 75	-	-	-	0.02	1.03
Unigene0090780	DNA-binding WRKY	-	-	-	3.30	−3.58
Unigene0094842	Probable WRKY transcription factor 53	-	-	-	1.26	0.40
Unigene0098001	WRKY transcription factor 5, partial	-	-	-	0.00	0.58
Unigene0101998	Probable WRKY transcription factor 49	-	-	-	−0.17	1.72
Unigene0104865	WRKY transcription factor 8	-	-	-	0.11	0.29
Unigene0104866	WRKY transcription factor 8	-	-	-	0.96	0.44

Note: N-48 h: 200 mM NaCl-48 h, N + K-48 h:200 mM NaCl + 10 mM KCl-48 h, N-168 h: 200 mM NaCl-168 h, N + K-168 h:200 mM NaCl + 10 mM KCl-168 h; ko04626: plant-interaction pathway, ko04016: MAPK signaling pathway-plant pathway.

Supplementary Table S3. Predicted data table of Pfam protein structural domain of candidate key *WRKY* genes

Gene ID	envelope start	envelope end	hmm acc	hmm length	bit score	clan	PfamA_definition
Unigene0000276	181	239	PF03106.15	59	82.5	CL0274	WRKY DNA -binding domain
Unigene0001294	102	122	PF03106.15	59	30.1	CL0274	WRKY DNA -binding domain
Unigene0001715	330	388	PF03106.15	59	82.5	CL0274	WRKY DNA -binding domain
Unigene0004936	102	159	PF03106.15	59	88.1	CL0274	WRKY DNA -binding domain
Unigene0007135	244	301	PF03106.15	59	87.9	CL0274	WRKY DNA -binding domain
Unigene0007190	130	190	PF03106.15	59	85.4	CL0274	WRKY DNA -binding domain
Unigene0009588	-	-	-	-	-	-	-
Unigene0010090	208	265	PF03106.15	59	87.1	CL0274	WRKY DNA -binding domain
Unigene0010425	132	152	PF03106.15	59	27.3	CL0274	WRKY DNA -binding domain
Unigene0011343	161	218	PF03106.15	59	92.1	CL0274	WRKY DNA -binding domain
Unigene0012574	30	52	PF03106.15	59	23.7	CL0274	WRKY DNA -binding domain
Unigene0014405	21	79	PF03106.15	59	84.8	CL0274	WRKY DNA -binding domain
Unigene0012609	61	118	PF03106.15	59	85.8	CL0274	WRKY DNA -binding domain
Unigene0016411	186	243	PF03106.15	59	75.1	CL0274	WRKY DNA -binding domain
Unigene0016969	-	-	-	-	-	-	-
Unigene0017105	221	277	PF03106.15	59	83.2	CL0274	WRKY DNA -binding domain
Unigene0020906	123	183	PF03106.15	59	90.6	CL0274	WRKY DNA -binding domain
Unigene0020962	176	233	PF03106.15	59	88.0	CL0274	WRKY DNA -binding domain
Unigene0021960	89	147	PF03106.15	59	87.7	CL0274	WRKY DNA -binding domain
Unigene0023528	-	-	-	-	-	-	-
Unigene0024962	221	278	PF03106.15	59	92.8	CL0274	WRKY DNA -binding domain
Unigene0024963	44	100	PF03106.15	59	82.6	CL0274	WRKY DNA -binding domain
Unigene0025290	117	174	PF03106.15	59	94.3	CL0274	WRKY DNA -binding domain
Unigene0027474	315	373	PF03106.15	59	91.5	CL0274	WRKY DNA -binding domain
Unigene0032744	203	261	PF03106.15	59	85.5	CL0274	WRKY DNA -binding domain

Unigene0033084	225	281	PF03106.15	59	88.9	CL0274	WRKY DNA -binding domain
Unigene0034483	113	170	PF03106.15	59	86.3	CL0274	WRKY DNA -binding domain
Unigene0041792	32	89	PF03106.15	59	96.4	CL0274	WRKY DNA -binding domain
Unigene0045882	262	320	PF03106.15	59	84.3	CL0274	WRKY DNA -binding domain
Unigene0051904	293	334	PF03106.15	59	65.3	CL0274	WRKY DNA -binding domain
Unigene0052215	432	489	PF03106.15	59	86.5	CL0274	WRKY DNA -binding domain
Unigene0052366	-	-	-	-	-	-	-
Unigene0053897	174	232	PF03106.15	59	82.6	CL0274	WRKY DNA -binding domain
Unigene0055249	281	339	PF03106.15	59	85.9	CL0274	WRKY DNA -binding domain
Unigene0055838	122	182	PF03106.15	59	88.3	CL0274	WRKY DNA -binding domain
Unigene0061328	1	41	PF03106.15	59	52.7	CL0274	WRKY DNA -binding domain
Unigene0061329	300	360	PF03106.15	59	90.8	CL0274	WRKY DNA -binding domain
Unigene0062497	-	-	-	-	-	-	-
Unigene0065429	325	383	PF03106.15	59	89.2	CL0274	WRKY DNA -binding domain
Unigene0066131	86	114	PF03106.15	59	28.8	CL0274	WRKY DNA -binding domain
Unigene0068525	125	182	PF03106.15	59	87.4	CL0274	WRKY DNA -binding domain
Unigene0070215	30	88	PF03106.15	59	74.0	CL0274	WRKY DNA -binding domain
Unigene0075548	90	148	PF03106.15	59	88.3	CL0274	WRKY DNA -binding domain
Unigene0075549	1	39	PF03106.15	59	53.1	CL0274	WRKY DNA -binding domain
Unigene0076253	1	41	PF03106.15	59	49.3	CL0274	WRKY DNA -binding domain
Unigene0076920	188	248	PF03106.15	59	89.5	CL0274	WRKY DNA -binding domain
Unigene0077293	1	39	PF03106.15	59	51.0	CL0274	WRKY DNA -binding domain
Unigene0078678	158	218	PF03106.15	59	82.0	CL0274	WRKY DNA -binding domain
Unigene0078949	186	243	PF03106.15	59	92.5	CL0274	WRKY DNA -binding domain
Unigene0079313	238	296	PF03106.15	59	85.5	CL0274	WRKY DNA -binding domain
Unigene0079542	315	371	PF03106.15	59	88.9	CL0274	WRKY DNA -binding domain

Unigene0079543	101	156	PF03106.15	59	76.9	CL0274	WRKY DNA -binding domain
Unigene0083814	287	345	PF03106.15	59	90.8	CL0274	WRKY DNA -binding domain
Unigene0088230	136	196	PF03106.15	59	82.6	CL0274	WRKY DNA -binding domain
Unigene0090616	52	109	PF03106.15	59	92.3	CL0274	WRKY DNA -binding domain
Unigene0090780	3	61	PF03106.15	59	81.4	CL0274	WRKY DNA -binding domain
Unigene0094842	145	205	PF03106.15	59	89.4	CL0274	WRKY DNA -binding domain
Unigene0098001	379	435	PF03106.15	59	88.3	CL0274	WRKY DNA -binding domain
Unigene0101998	137	194	PF03106.15	59	88.2	CL0274	WRKY DNA -binding domain
Unigene0104865	381	438	PF03106.15	59	88.9	CL0274	WRKY DNA -binding domain
Unigene0104866	394	451	PF03106.15	59	86.0	CL0274	WRKY DNA -binding domain

Note: envelope start: HMM model predicted structural domain start position of Unigene coding protein sequence, envelope end: HMM model predicted structural domain end position of Unigene coding protein sequence, hmm acc: Unigene encodes the number of the HMM corresponding to the structure of the protein sequence in Pfam, hmm length: length of matching sequence in the database, bit score: score of Unigene coding protein sequence structure compared to HMM model, clan: classification of Unigene coding protein sequence by protein sequence, structure and HMM file in the Pfam database, PfamA_definition: the name of the structure corresponding to the query sequence in PfamA.

Supplementary Table S4. Information sheet for 15 species

Family	Species	Description	Gene	Protein ID	CDS (bp)	ORF length (aa)
Tamaricaceae	*Tamarix hispida*	WRKY transcription factor 1	*ThWRKY1*	AFS64067.1	1500	499
Amaranthaceae	*Beta vulgaris* subsp. *vulgaris*	WRKY transcription factor 1	*BvWRKY1*	XP_010666094.1	1326	441
Camellia lanceoleosa	*Camellia lanceoleosa*	WRKY transcription factor 1	*ClWRKY1*	KAI8007490.1	1407	468
Amaranthaceae	*Spinacia oleracea*	WRKY transcription factor 1	*SoWRKY1*	XP_021865070.1	1314	437
Lythraceae	*Punica granatum*	WRKY transcription factor 1	*PgWRKY1*	XP_031382379.1	1521	506
Theaceae	*Camellia sinensis*	WRKY transcription factor 1	*CsWRKY1*	AYA73382.1	1407	468
Vitis riparia	*Vitis riparia*	probable WRKY transcription factor 20	*VrWRKY20*	XP_034700767.1	1224	407
Euphorbiaceae	*Manihot esculenta*	WRKY transcription factor 29	*MeWRKY29*	AMO00397.1	1299	432
Malvaceae	*Gossypium hirsutum*	WRKY transcription factor 1	*GhWRKY1*	XP_016746117.1	1329	442
Juglandaceae	*Juglans regia*	WRKY transcription factor 1	*JrWRKY1*	XP_018824691.1	1446	481
Proteaceae	*Macadamia integrifolia*	probable WRKY transcription factor 20	*MiWRKY20*	XP_042490514.1	1935	644
Actinidiaceae	*Actinidia chinensis* var. *chinensis*	WRKY transcription factor 20	*AcWRKY20*	PSS16095.1	1473	490
Juglandaceae	*Carya illinoinensis*	WRKY transcription factor 1	*CiWRKY1*	XP_042974499.1	1395	464
Caricaceae	*Carica papaya*	WRKY transcription factor 1	*CpWRKY1*	XP_021889975.1	1167	388
Malvaceae	*Hibiscus syriacus*	WRKY transcription factor 1	*HsWRKY1*	KAE8709287.1	1158	385

Note: CDS: CoDing Sequence; ORF: Open Reading Frame.

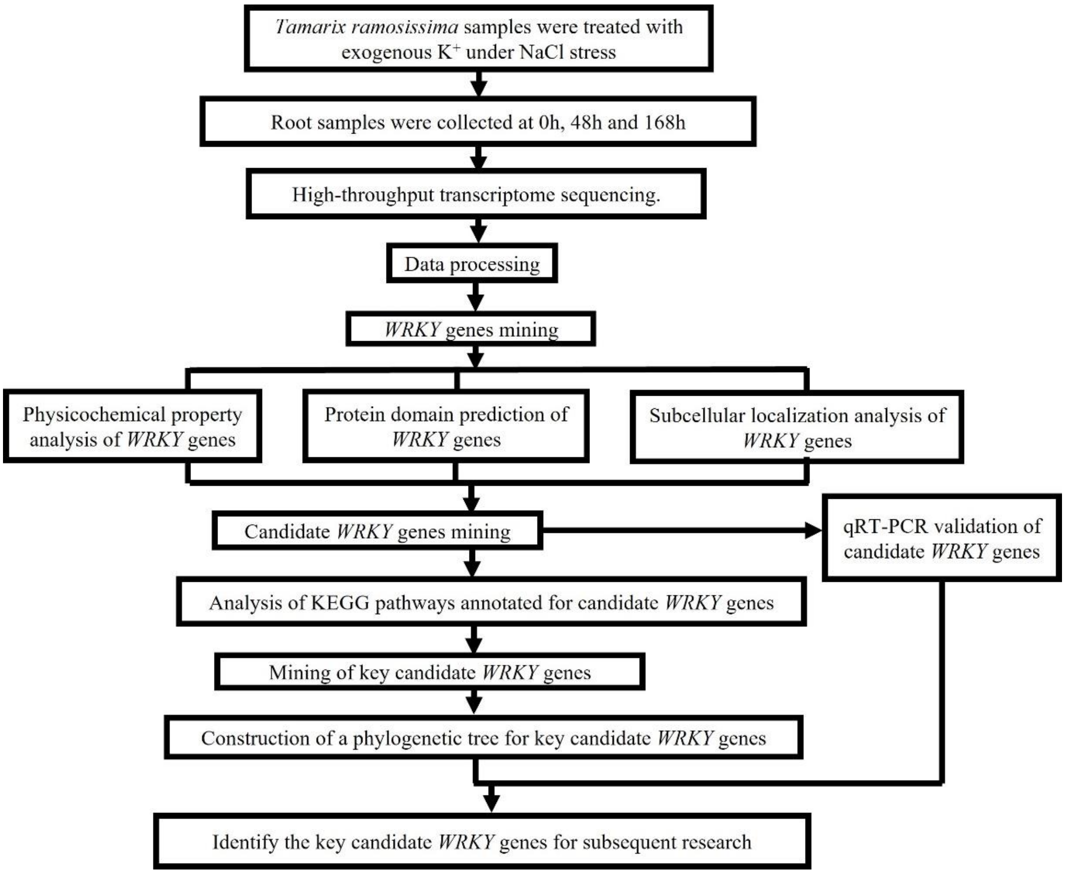

Supplementary Figure S1. Experimental technical flowchart

(This figure provides a detailed overview of the technical approach in this manuscript.).

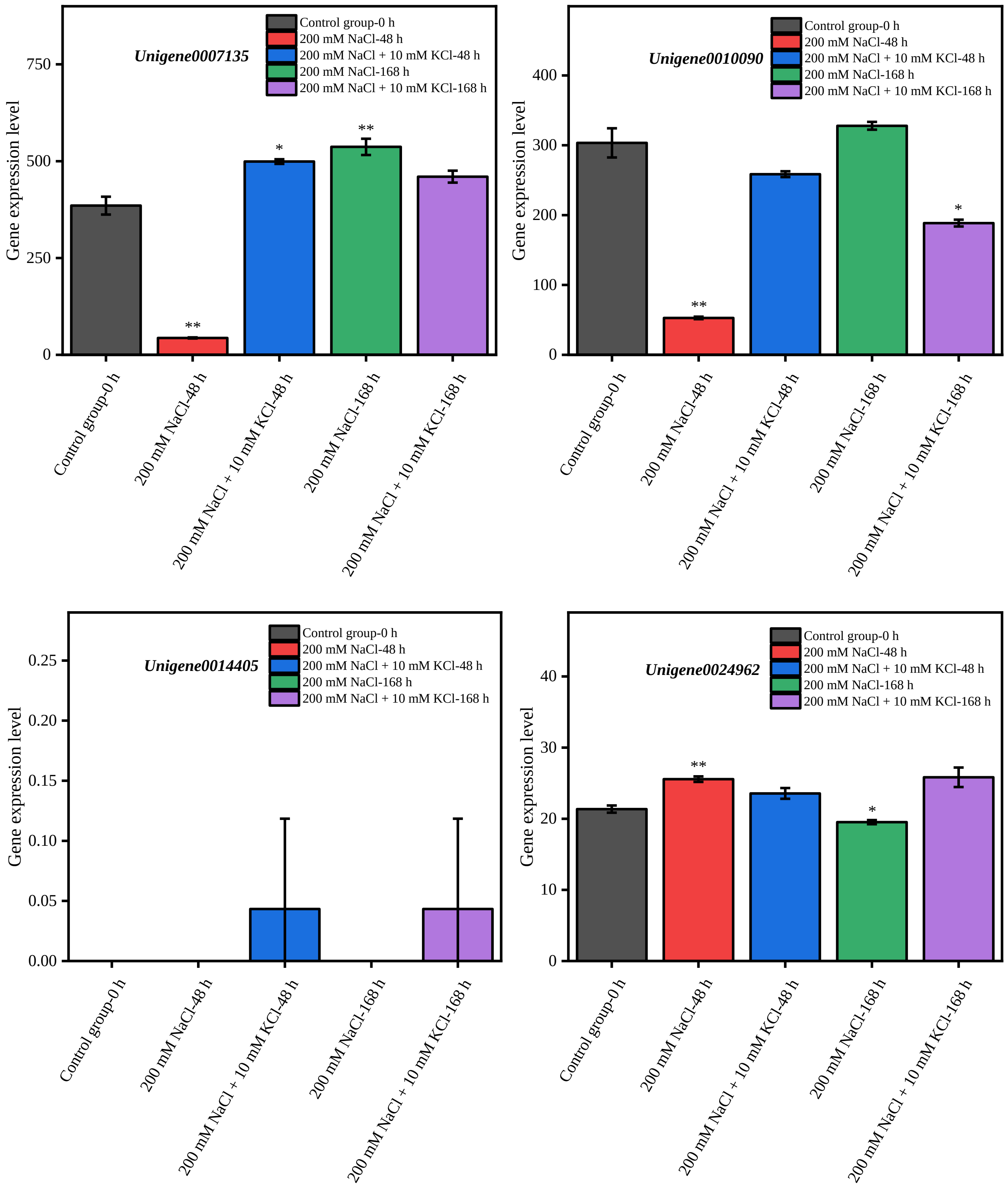
Unigene0007135
Unigene0010090
Unigene0014405
Unigene0024962
Control group-0 h
200 mM NaCl-48 h
200 mM NaCl + 10 mM KCl-48 h
200 mM NaCl-168 h
200 mM NaCl + 10 mM KCl-168 h
Gene expression level
0
250
500
750
100
200
300
400
0.00
0.05
0.10
0.15
0.20
0.25
10
20
30
40
*
**

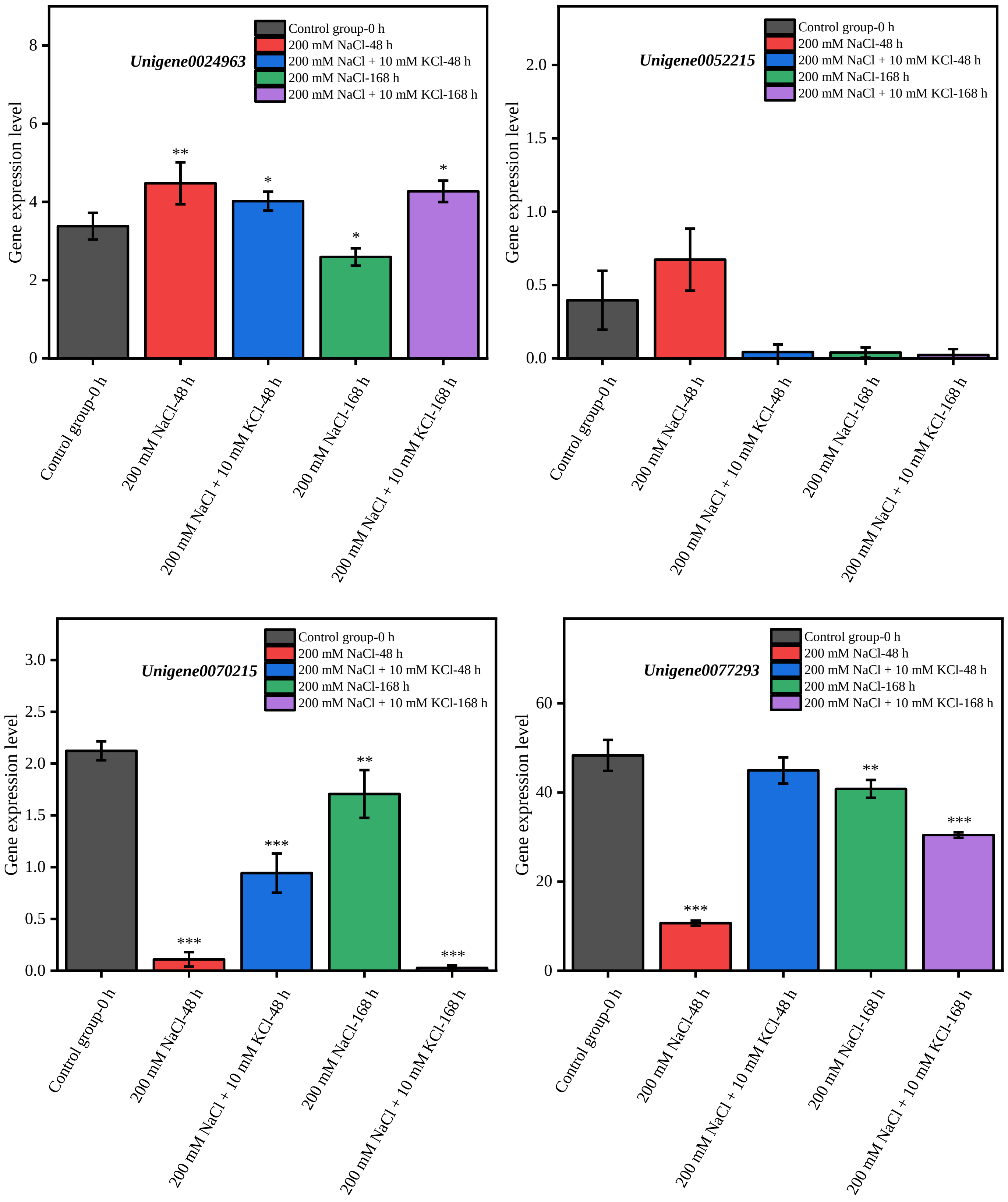
Unigene0024963
Unigene0052215
Unigene0070215
Unigene0077293
Gene expression level
Control group-0 h
200 mM NaCl-48 h
200 mM NaCl + 10 mM KCl-48 h
200 mM NaCl-168 h
200 mM NaCl + 10 mM KCl-168 h

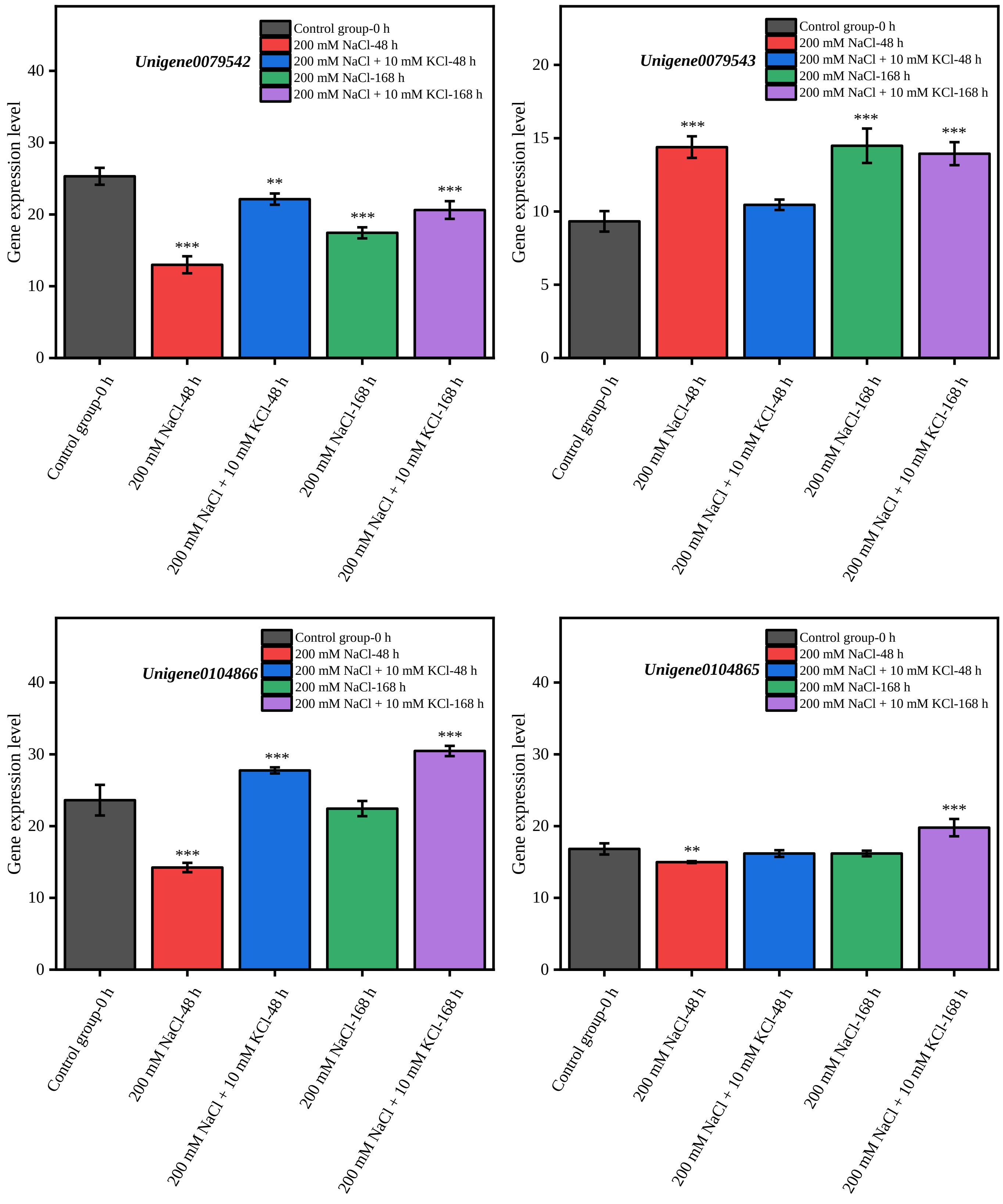

Unigene0079542
Unigene0079543
Unigene0104866
Unigene0104865
Control group-0 h
200 mM NaCl-48 h
200 mM NaCl + 10 mM KCl-48 h
200 mM NaCl-168 h
200 mM NaCl + 10 mM KCl-168 h
Gene expression level
0
5
10
15
20
30
40
**
***

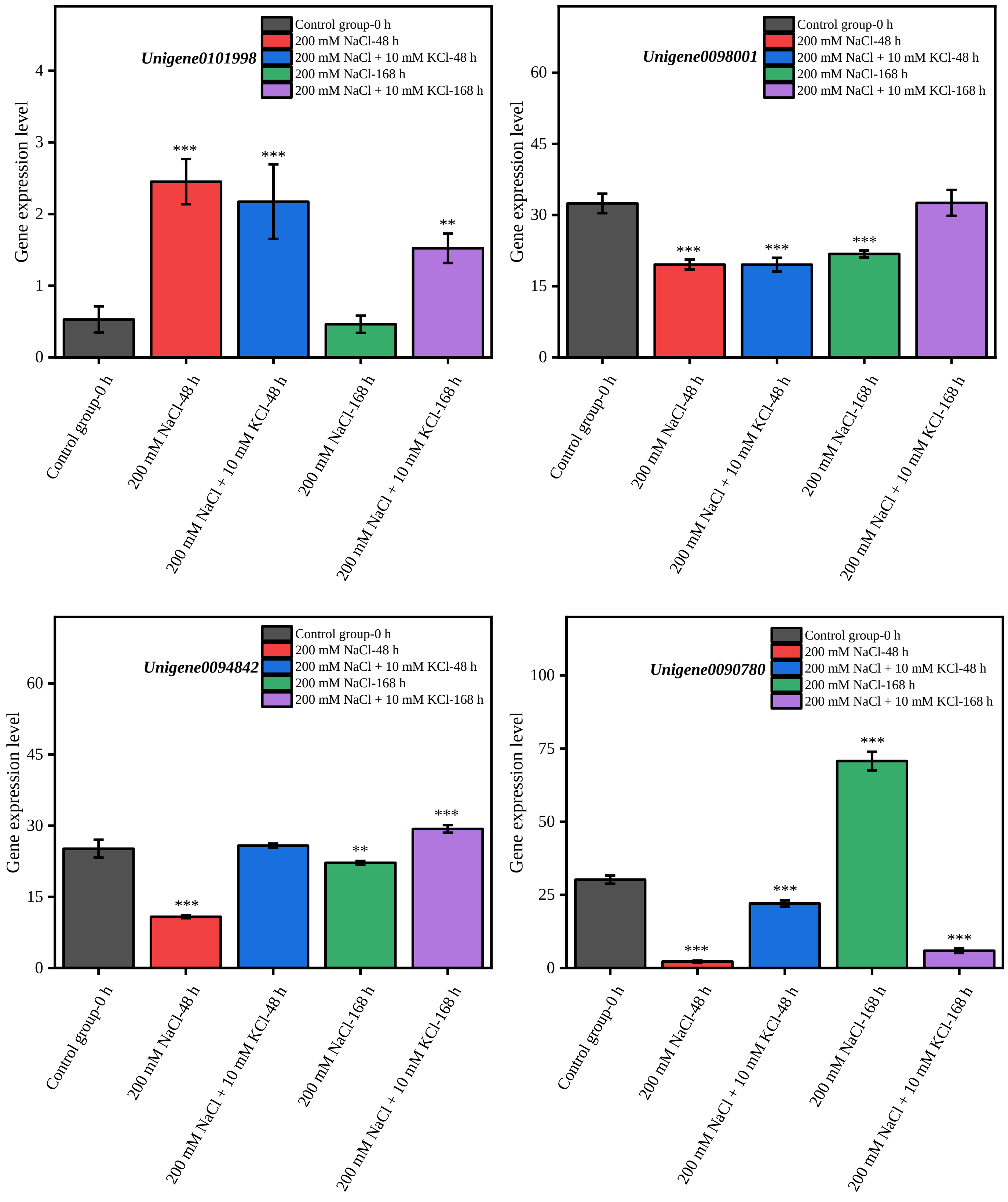
Unigene0101998
Unigene0098001
Unigene0094842
Unigene0090780
Control group-0 h
200 mM NaCl-48 h
200 mM NaCl + 10 mM KCl-48 h
200 mM NaCl-168 h
200 mM NaCl + 10 mM KCl-168 h
Gene expression level
0
1
2
3
4
15
30
45
60
25
50
75
100
***
**

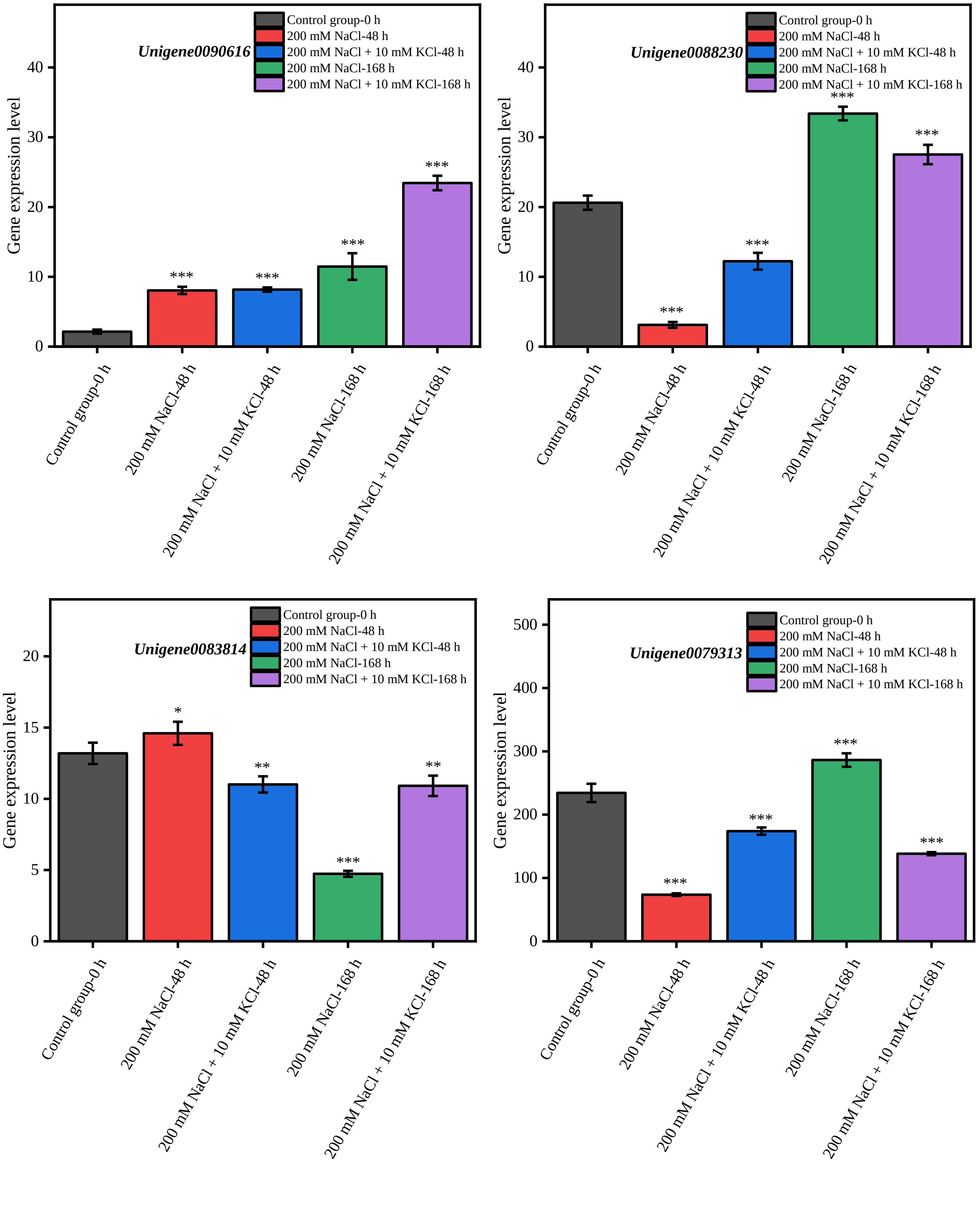
Unigene0090616
Unigene0088230
Unigene0083814
Unigene0079313
Control group-0 h
200 mM NaCl-48 h
200 mM NaCl + 10 mM KCl-48 h
200 mM NaCl-168 h
200 mM NaCl + 10 mM KCl-168 h
Gene expression level
***
**
*

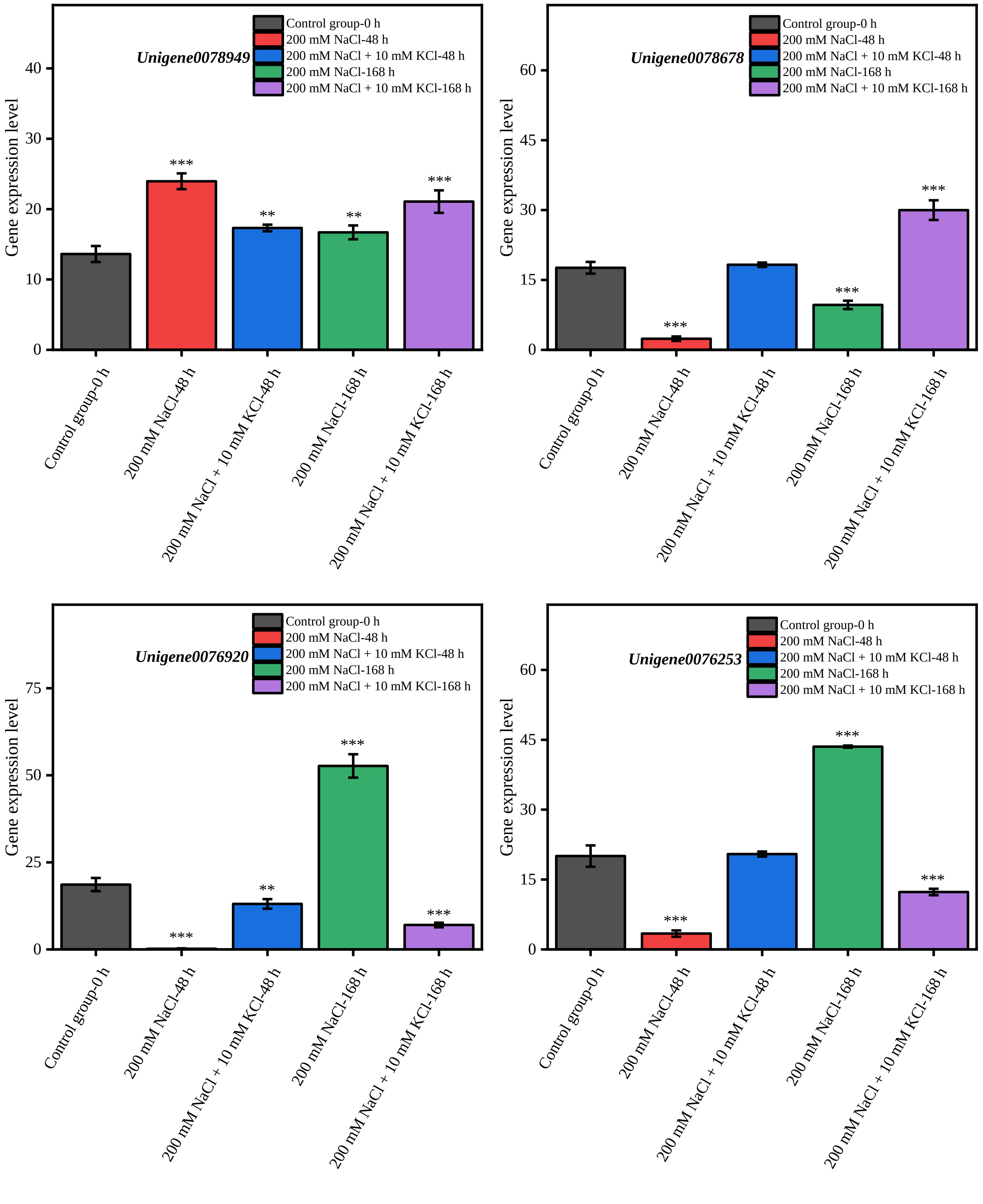
Unigene0078949
Unigene0078678
Unigene0076920
Unigene0076253
Control group-0 h
200 mM NaCl-48 h
200 mM NaCl + 10 mM KCl-48 h
200 mM NaCl-168 h
200 mM NaCl + 10 mM KCl-168 h
Gene expression level
0
10
20
30
40
15
45
60
25
50
75
***
**

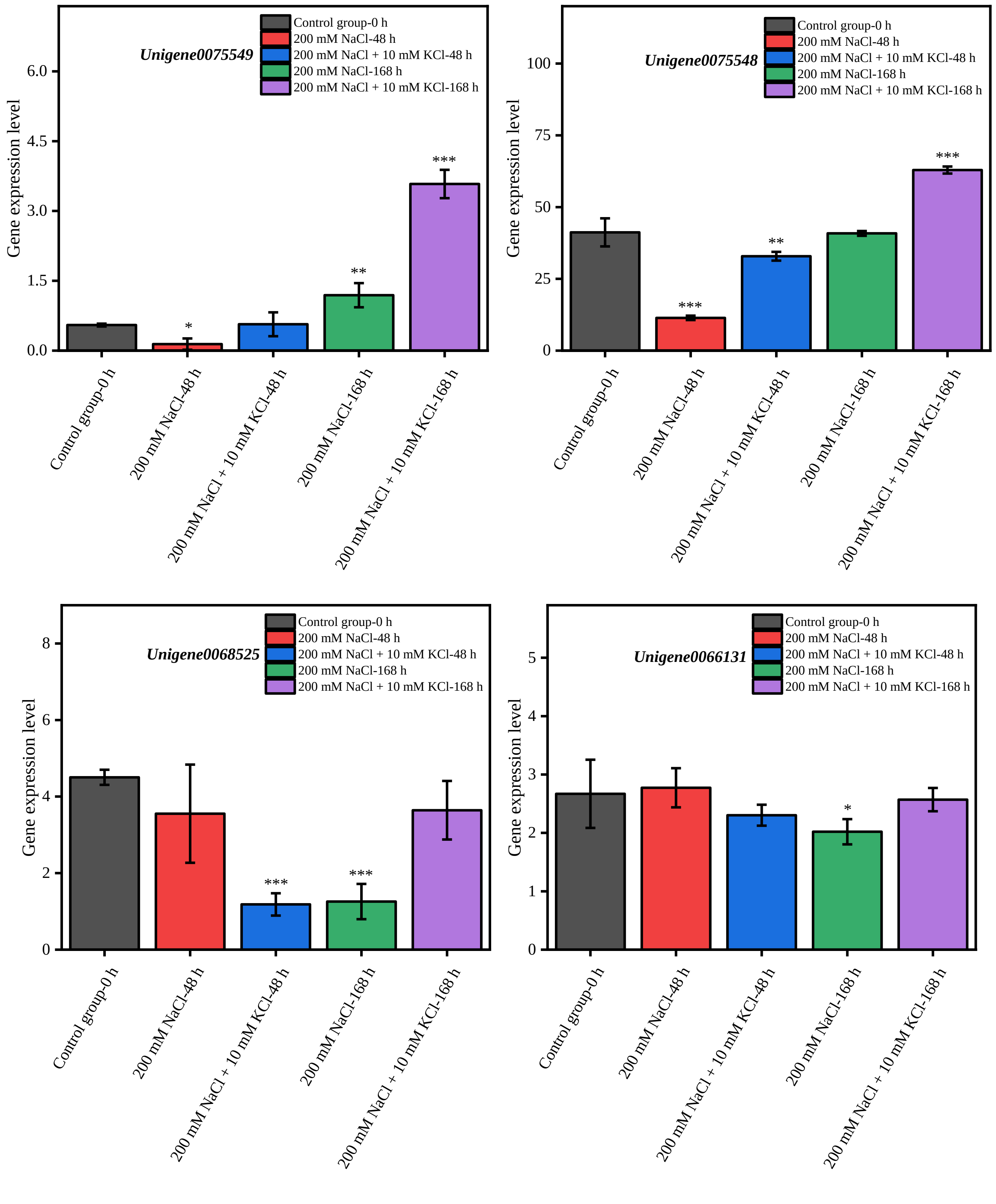
Unigene0075549
Unigene0075548
Unigene0068525
Unigene0066131
Gene expression level
Control group-0 h
200 mM NaCl-48 h
200 mM NaCl + 10 mM KCl-48 h
200 mM NaCl-168 h
200 mM NaCl + 10 mM KCl-168 h
0.0
1.5
3.0
4.5
6.0
0
25
50
75
100
2
4
6
8
1
3
5
*
**
***

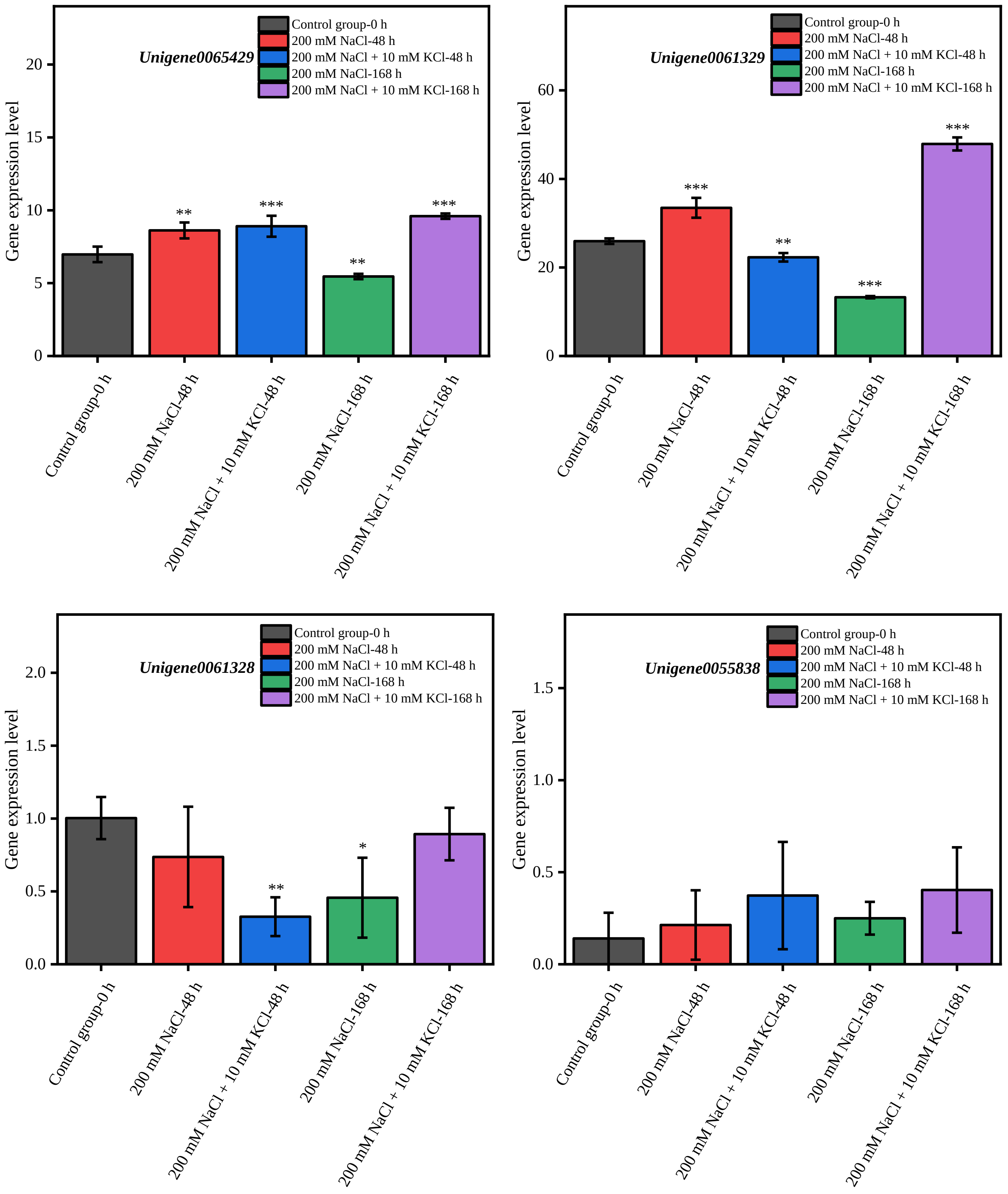
Unigene0065429
Unigene0061329
Unigene0061328
Unigene0055838
Gene expression level
Control group-0 h
200 mM NaCl-48 h
200 mM NaCl + 10 mM KCl-48 h
200 mM NaCl-168 h
200 mM NaCl + 10 mM KCl-168 h
**
***
*

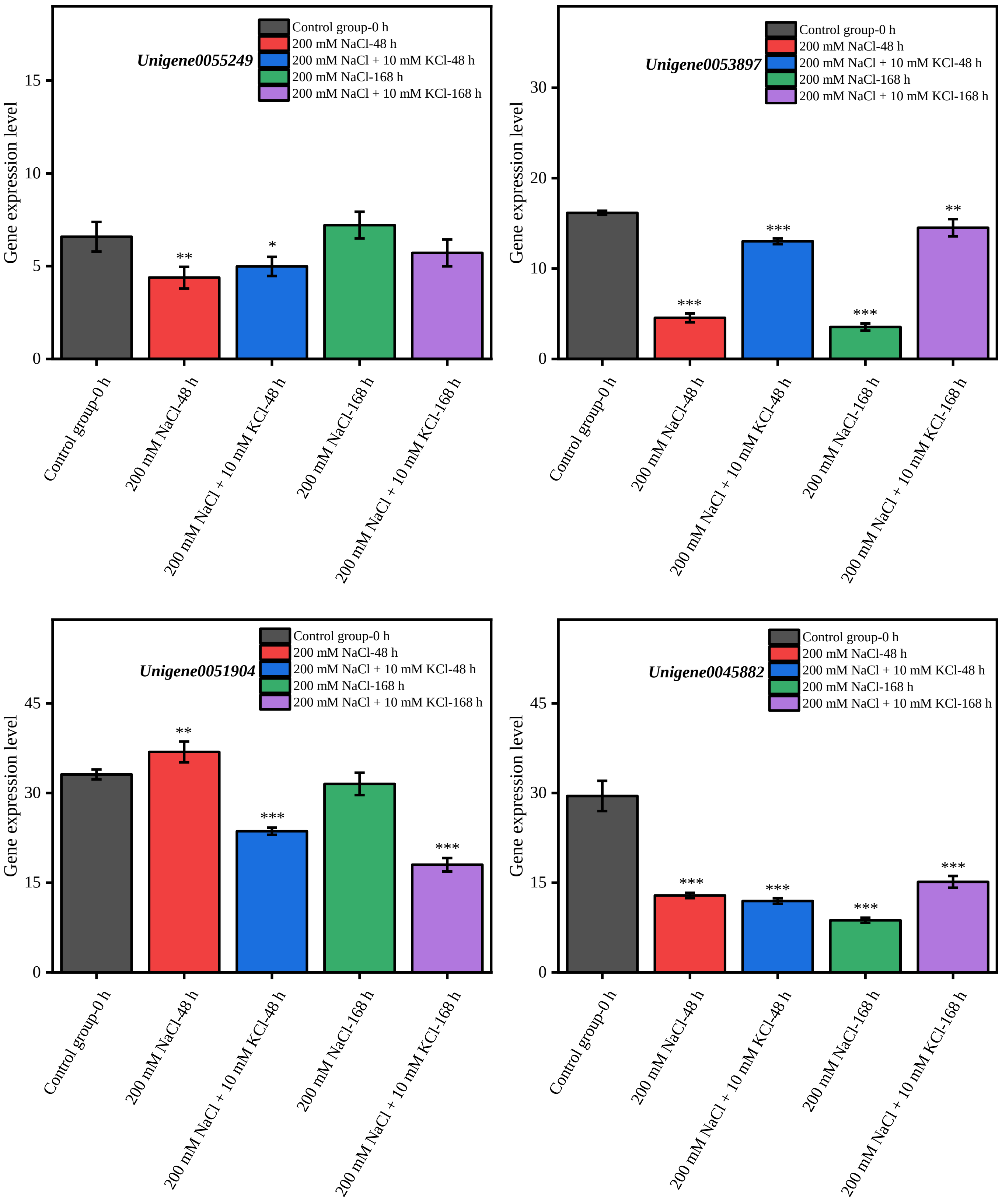
Unigene0055249
Unigene0053897
Unigene0051904
Unigene0045882
Control group-0 h
200 mM NaCl-48 h
200 mM NaCl + 10 mM KCl-48 h
200 mM NaCl-168 h
200 mM NaCl + 10 mM KCl-168 h
Gene expression level
0
5
10
15
20
30
45
**
*
***

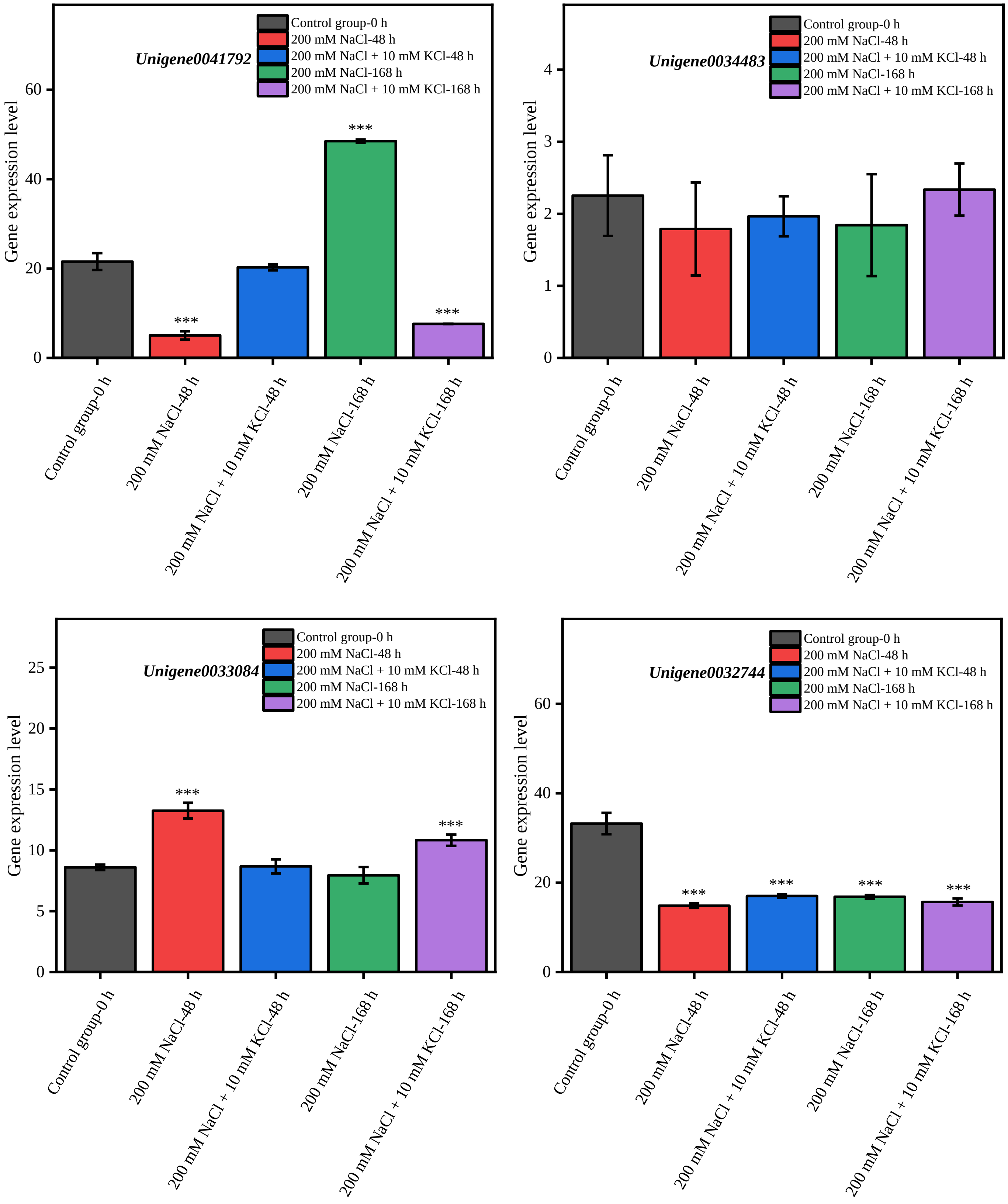
Unigene0041792
Unigene0034483
Unigene0033084
Unigene0032744
Gene expression level
Control group-0 h
200 mM NaCl-48 h
200 mM NaCl + 10 mM KCl-48 h
200 mM NaCl-168 h
200 mM NaCl + 10 mM KCl-168 h
***

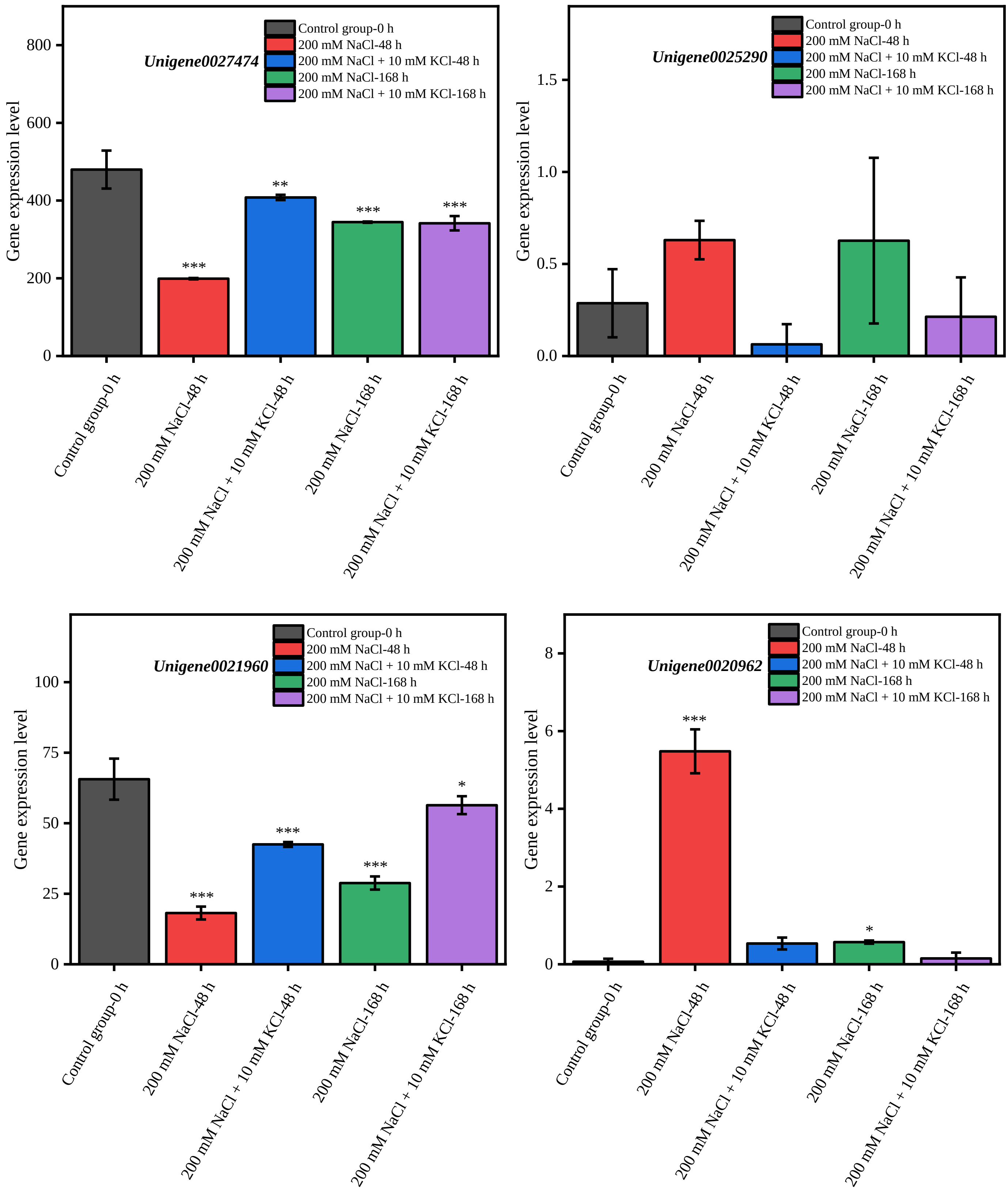

Unigene0027474
Unigene0025290
Unigene0021960
Unigene0020962
Control group-0 h
200 mM NaCl-48 h
200 mM NaCl + 10 mM KCl-48 h
200 mM NaCl-168 h
200 mM NaCl + 10 mM KCl-168 h
Gene expression level
0
200
400
600
800
0.0
0.5
1.0
1.5
25
50
75
100
2
4
6
8
***
**
*

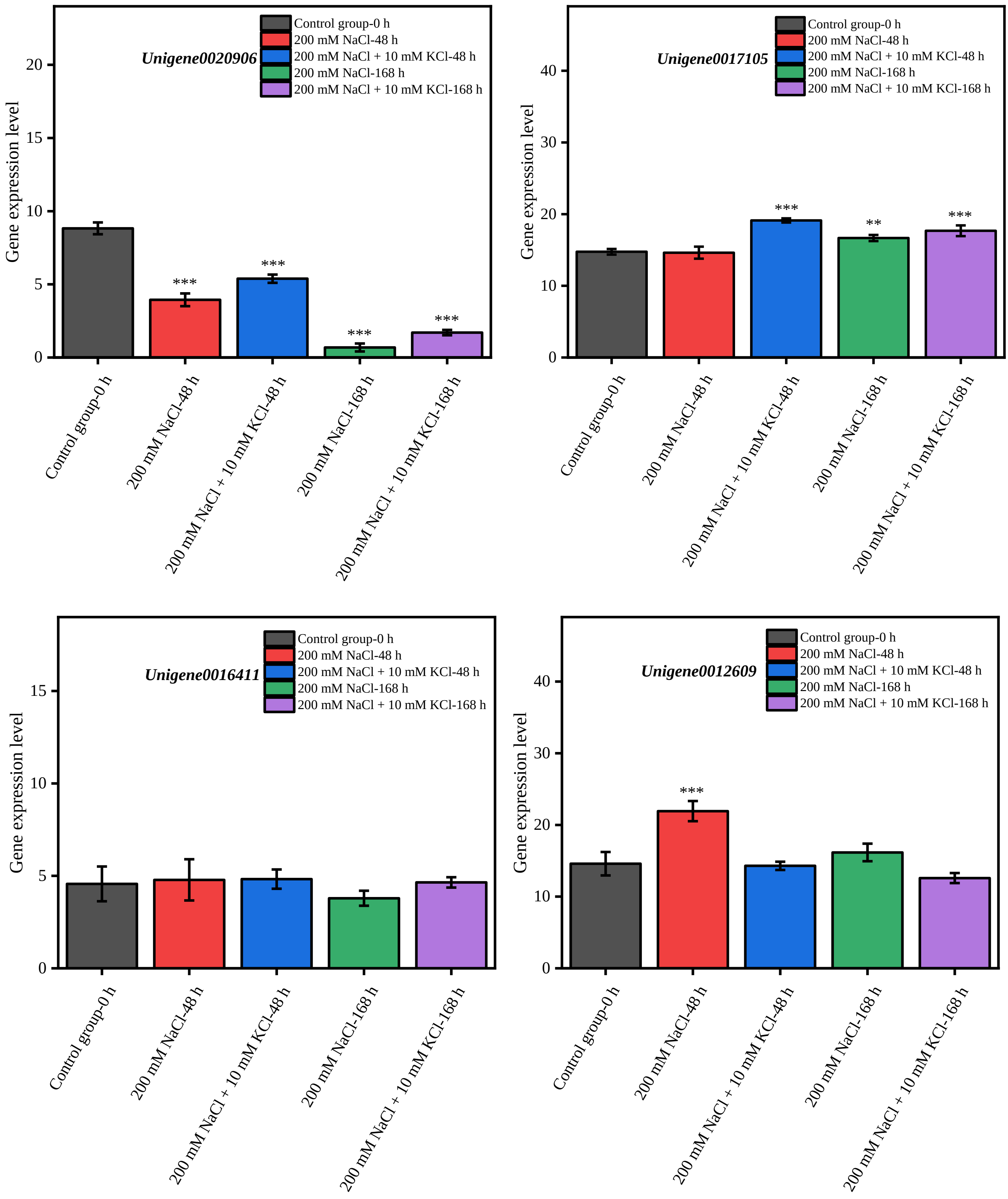
Unigene0020906
Unigene0017105
Unigene0016411
Unigene0012609
Control group-0 h
200 mM NaCl-48 h
200 mM NaCl + 10 mM KCl-48 h
200 mM NaCl-168 h
200 mM NaCl + 10 mM KCl-168 h
Gene expression level
0
5
10
15
20
30
40
***
**

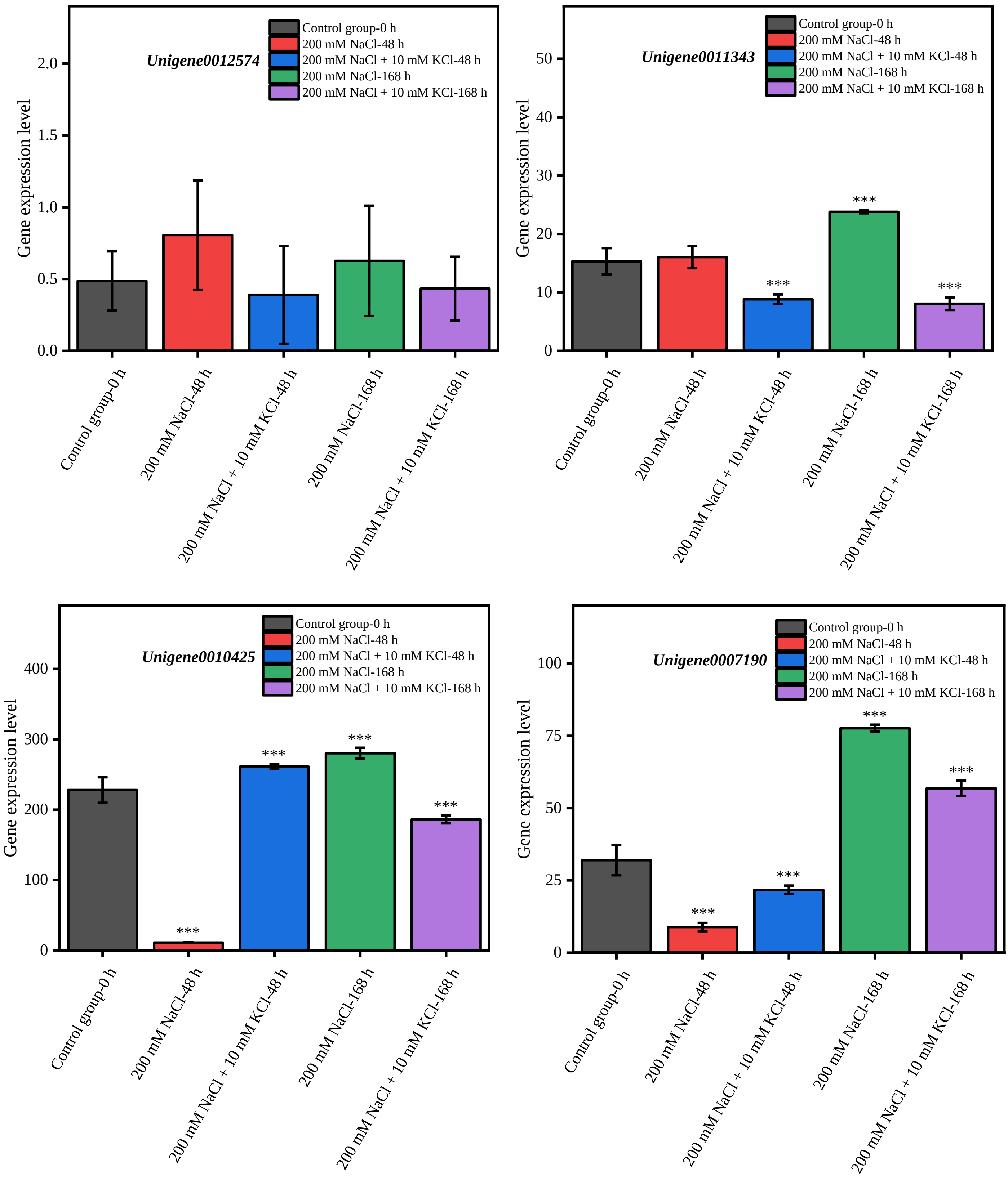
Unigene0012574
Unigene0011343
Unigene0010425
Unigene0007190
Control group-0 h
200 mM NaCl-48 h
200 mM NaCl + 10 mM KCl-48 h
200 mM NaCl-168 h
200 mM NaCl + 10 mM KCl-168 h
Gene expression level
***

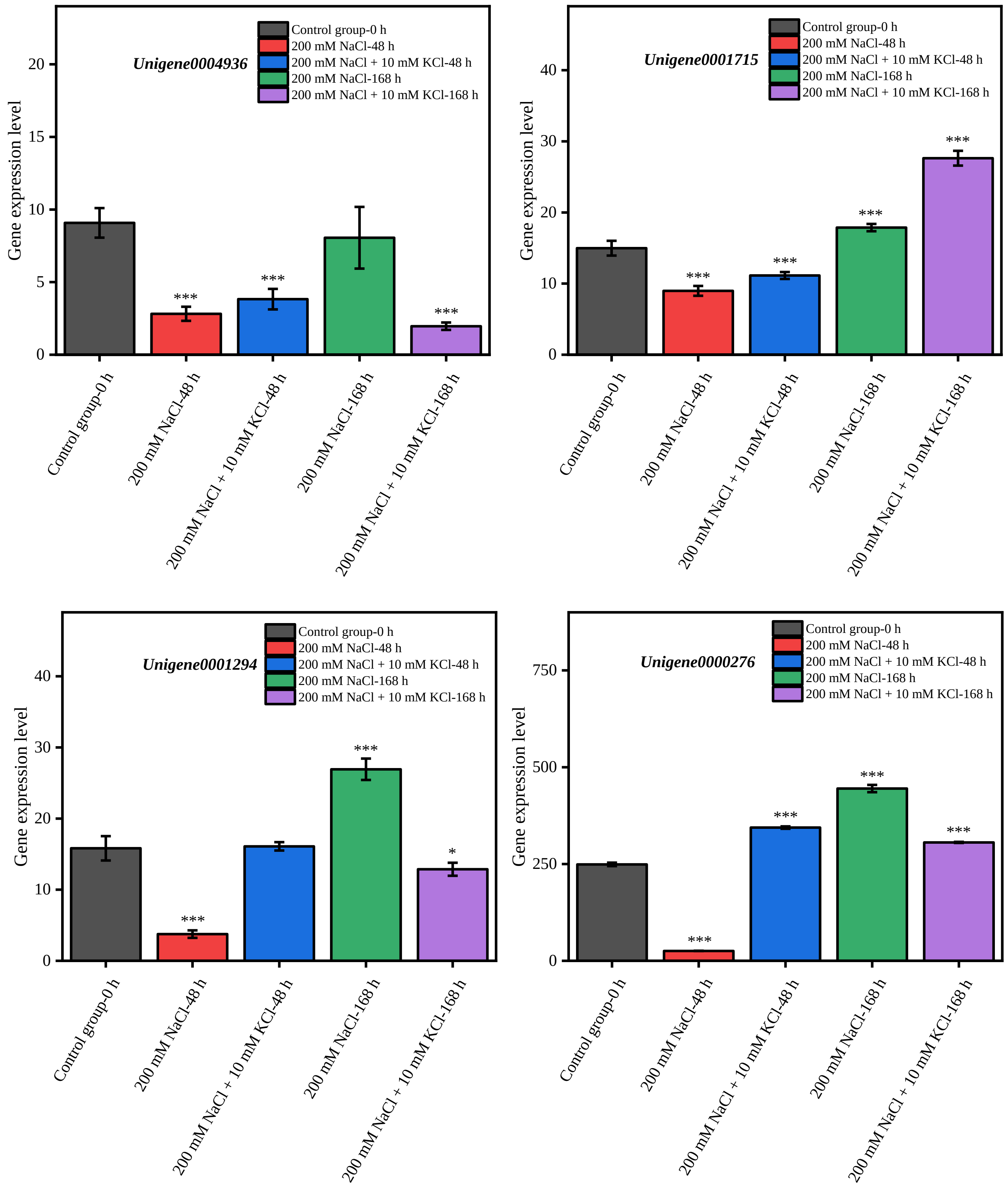

Supplementary Figure S2. Changes in the expression levels of *WRKY* genes in the roots of *T. ramosissima*
(After the application of exogenous NaCl stress for 48 and 168 hours on the *T. ramosissima*, the expression levels of 56 *WRKY* genes in its roots changed. Note: $p \geq 0.05$ is not marked; $0.01 < p < 0.05$ is marked as *; $0.001 < p < 0.01$ is marked as **; $p \leq 0.001$ is marked as ***)

200 mM NaCl-48 h vs. 200 mM NaCl + 10 mM KCl-48 h

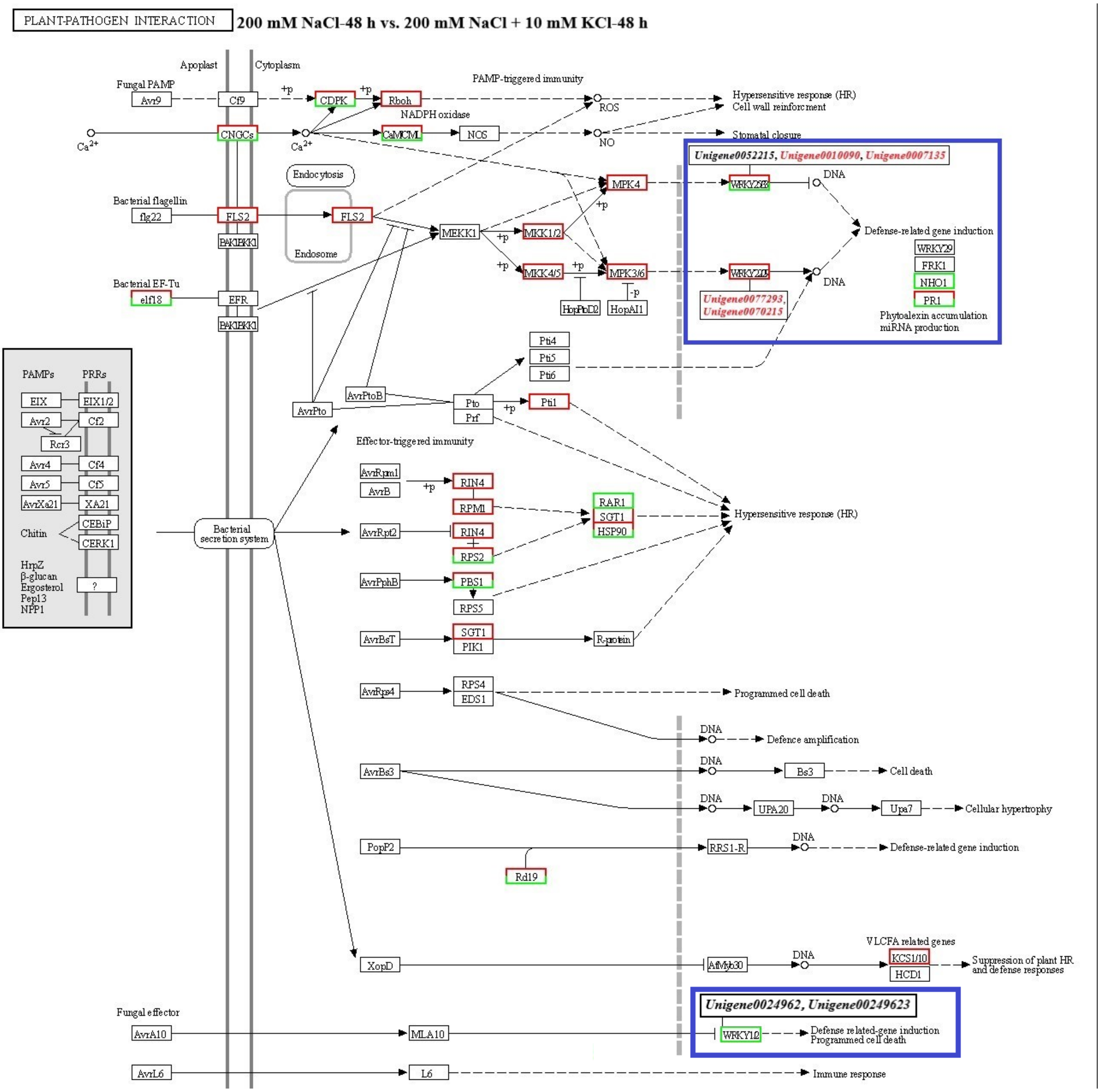

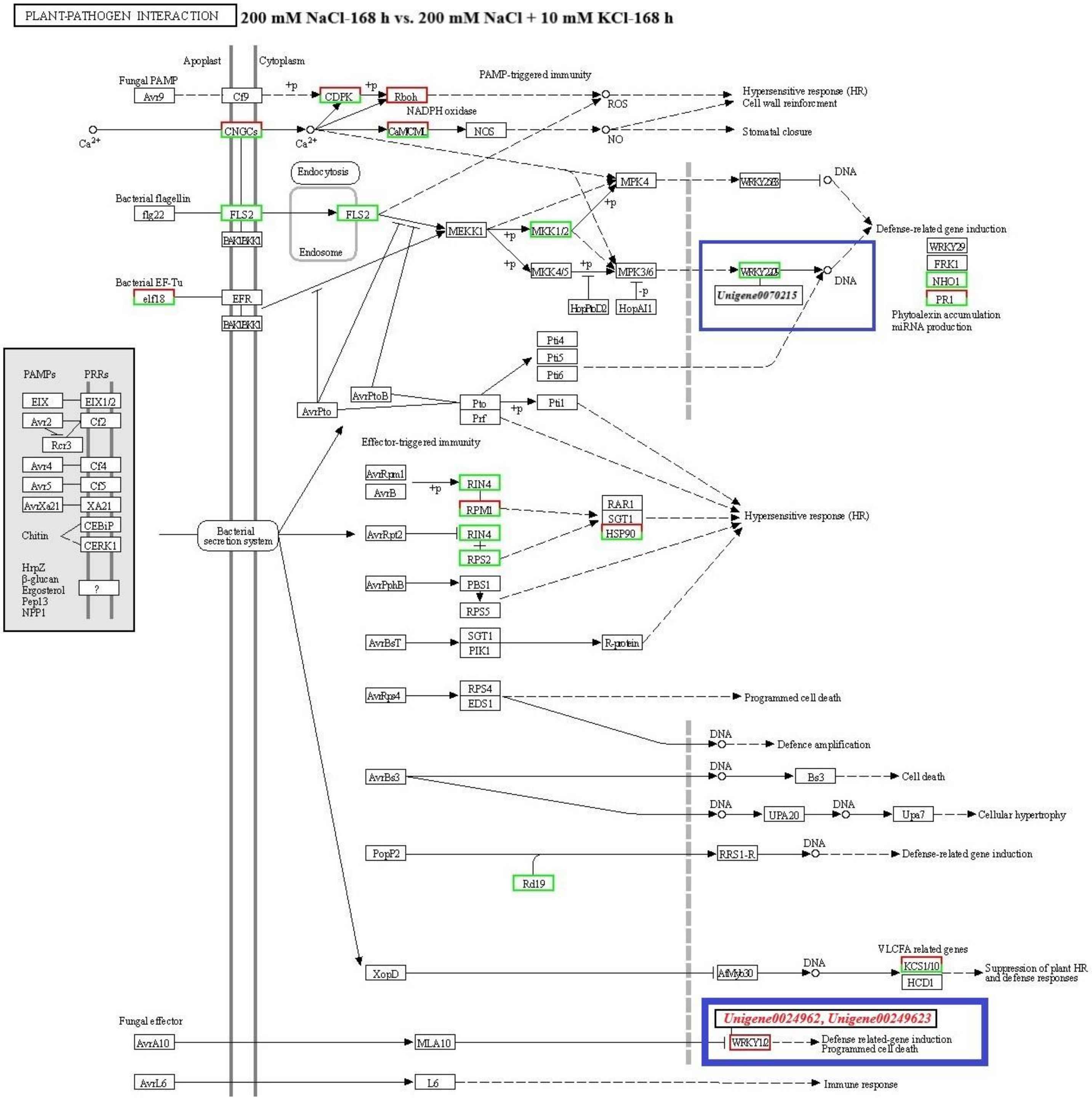

Supplementary Figure S3. Analysis of Plant-pathogen interaction pathway

(The DEGs were enriched into the Plant-pathogen interaction pathway when exogenous genes were applied for 48h and 168h under NaCl stress. The genes highlighted in the blue box belong to the *WRKY* gene. Note: Black marked genes: down-regulated genes; Genes in red: up-regulated genes. Red boxes in the figure: up-regulated genes exist; Green box in the figure: there are down-regulated genes; Red + green boxes in the figure: up-regulated and down-regulated genes exist).

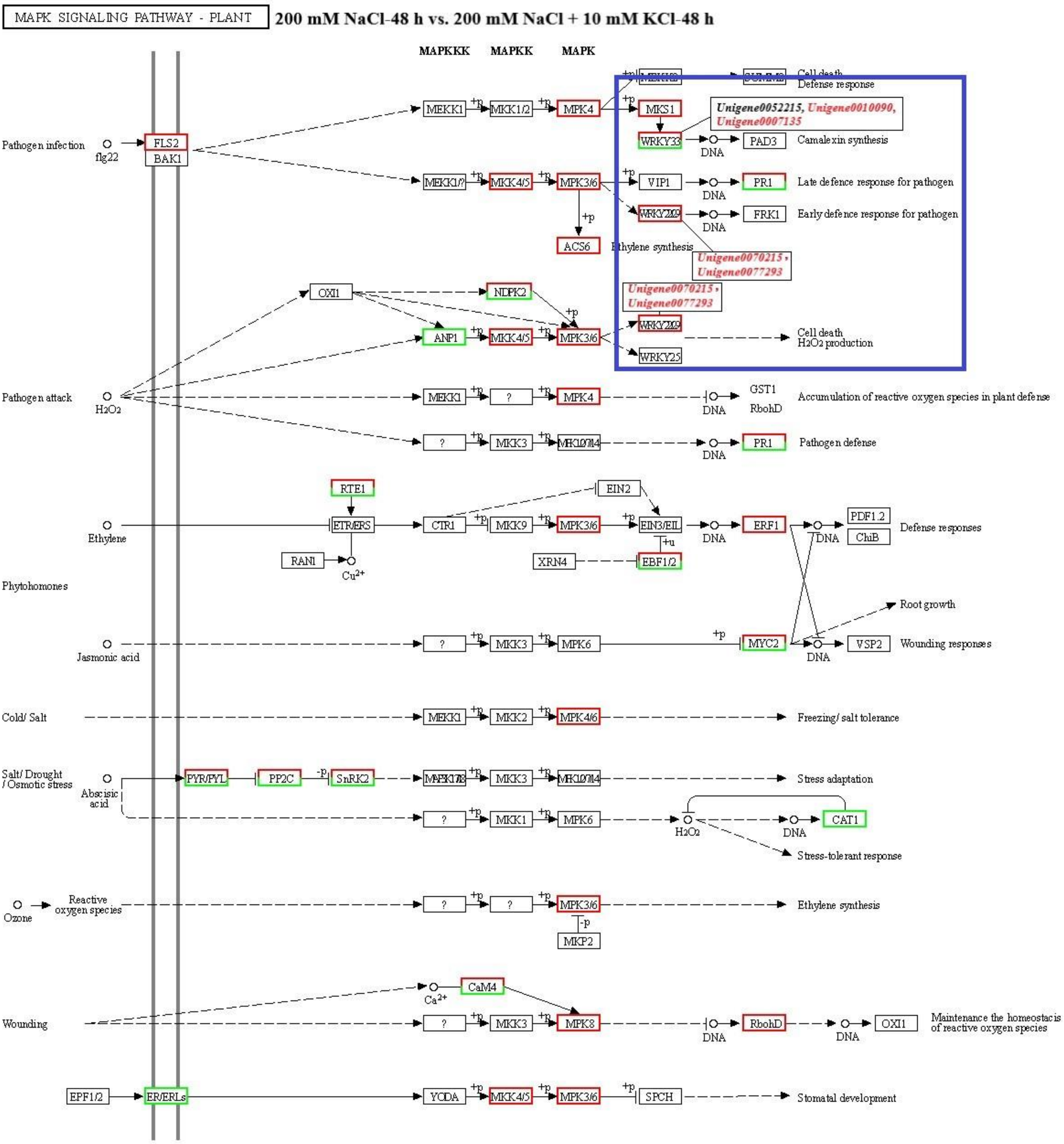

MAPK SIGNALING PATHWAY - PLANT
200 mM NaCl-48 h vs. 200 mM NaCl + 10 mM KCl-48 h
MAPKKK
MAPKK
MAPK
Pathogen infection
flg22
FLS2
BAK1
MEKK1
MKK1/2
MPK4
MKS1
WRKY33
PAD3
Camalexin synthesis
Cell death
Defense response
Unigene0052215, Unigene0010090, Unigene0007135
DNA
MEKK1/?
MKK4/5
MPK3/6
VIP1
PR1
Late defence response for pathogen
WRKY22/29
FRK1
Early defence response for pathogen
ACS6
Ethylene synthesis
Unigene0070215, Unigene0077293
OXI1
NDPK2
ANP1
WRKY22/29
WRKY25
Cell death
H2O2 production
Pathogen attack
H2O2
MPK4
GST1
RbohD
Accumulation of reactive oxygen species in plant defense
MKK3
MPK1/2/7/14
Pathogen defense
RTE1
EIN2
Ethylene
ETR/ERS
CTR1
MKK9
EIN3/EIL
ERF1
PDF1.2
ChiB
Defense responses
RAN1
Cu2+
XRN4
EBF1/2
Phytohomones
Root growth
Jasmonic acid
MPK6
MYC2
VSP2
Wounding responses
Cold/ Salt
MKK2
MPK4/6
Freezing/ salt tolerance
Salt/ Drought / Osmotic stress
Abscisic acid
PYR/PYL
PP2C
SnRK2
MAPKKK17/18
Stress adaptation
MKK1
CAT1
Stress-tolerant response
Ozone
Reactive oxygen species
MKP2
Ethylene synthesis
Wounding
Ca2+
CaM4
MPK8
OXI1
Maintenance the homeostacis of reactive oxygen species
EPF1/2
ER/ERLs
YODA
SPCH
Stomatal development

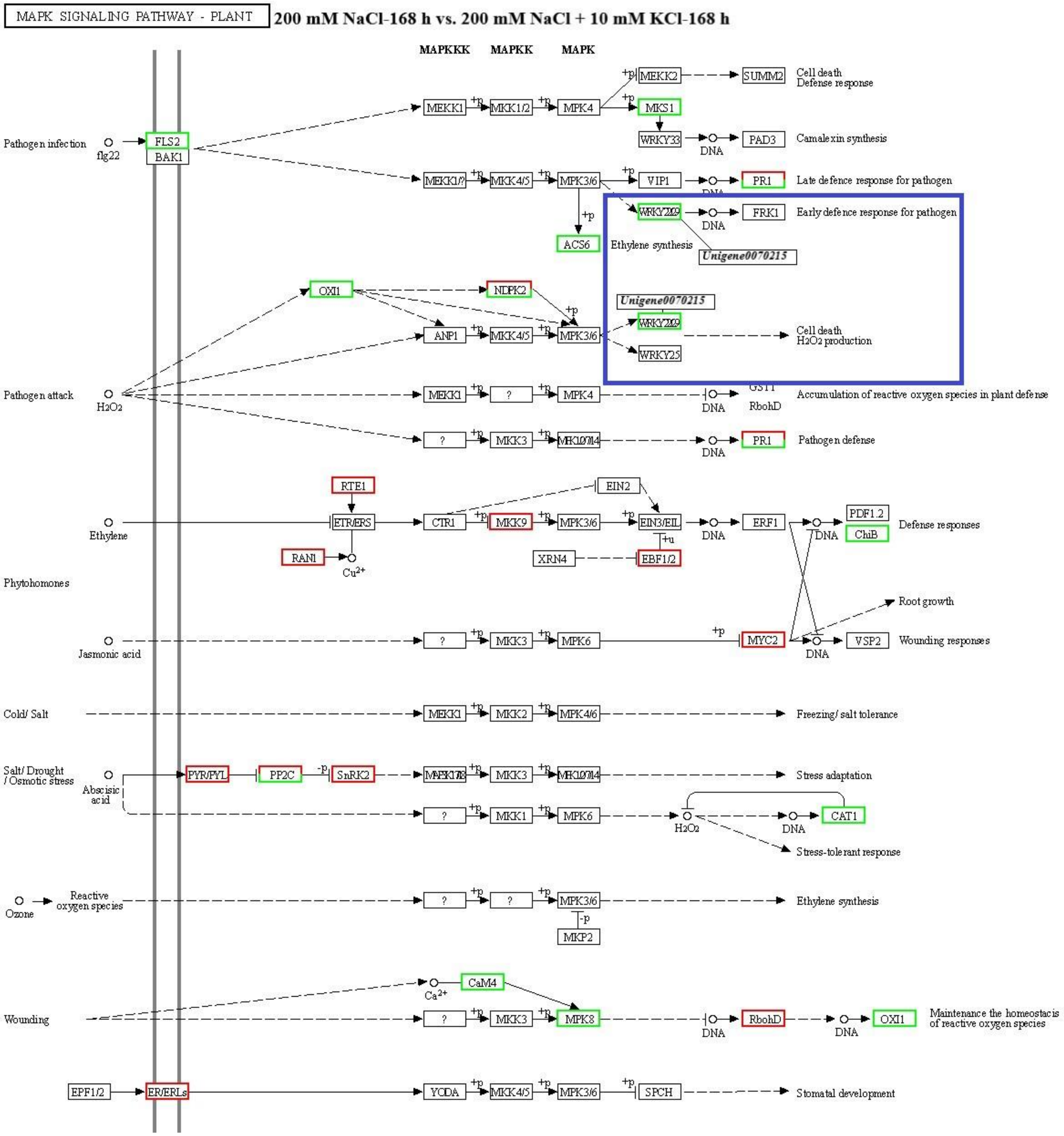

Supplementary Figure S4. Analysis of MAPK signaling pathway-plant pathway

(Differentially expressed genes were enriched to MAPK signaling pathway-plant pathway in 48h and 168h under NaCl stress. The genes highlighted in the blue box belong to the *WRKY* gene. Note: Genes marked in black: down-regulated genes; Genes in red: up-regulated genes. Red boxes in the figure: up-regulated genes exist; Green box in the figure: there are down-regulated genes; Red + green box in the figure: up-regulated and down-regulated genes exist).

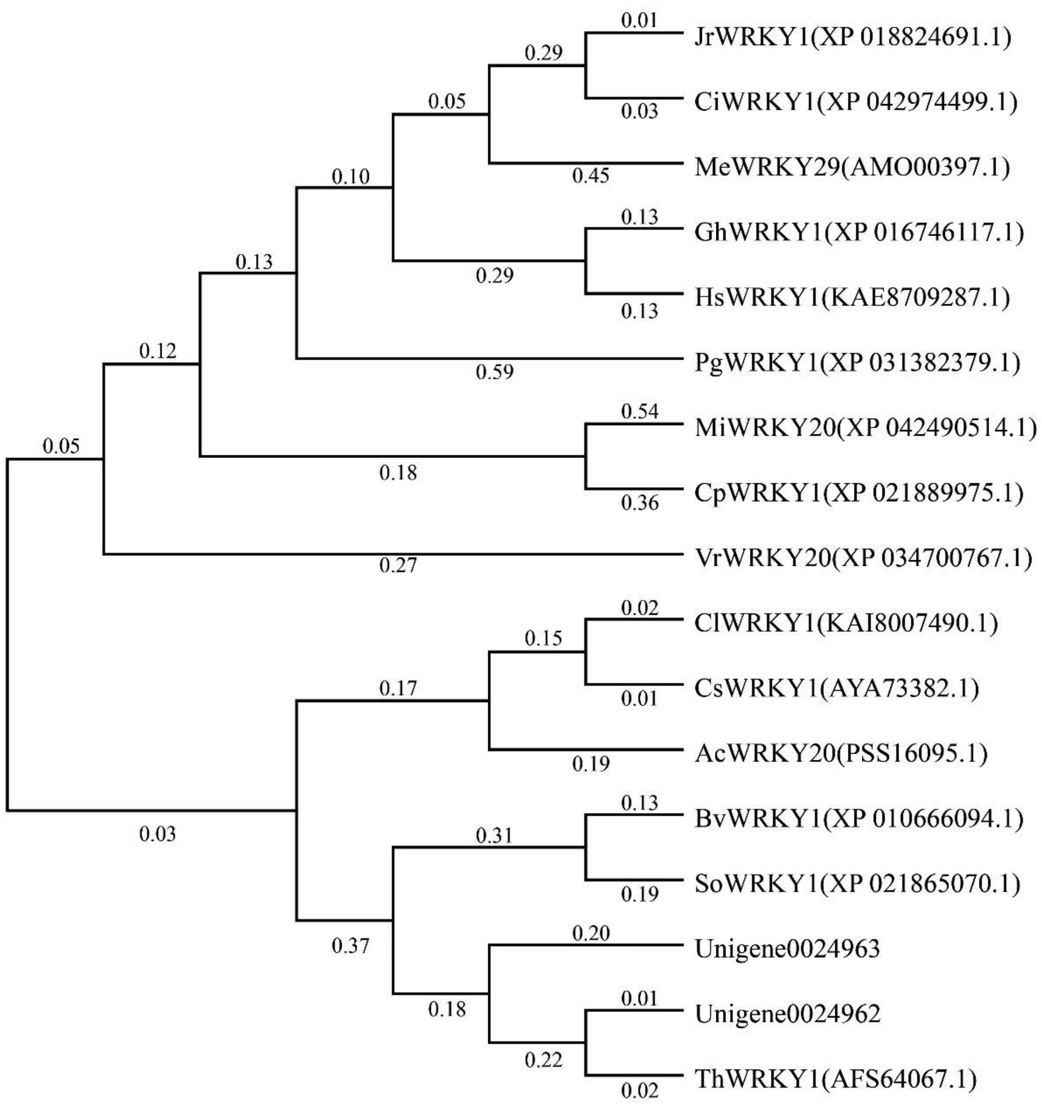

Supplementary Figure S5. Phylogenetic tree analysis of *T. ramosissima WRKY* gene and other species *WRKY* gene (Analyzing the phylogenetic tree constructed from the protein amino acid sequences of Unigene0024962 and Unigene0024963 from *T. ramosissima* roots, along with those of 15 other homologous gene species.).

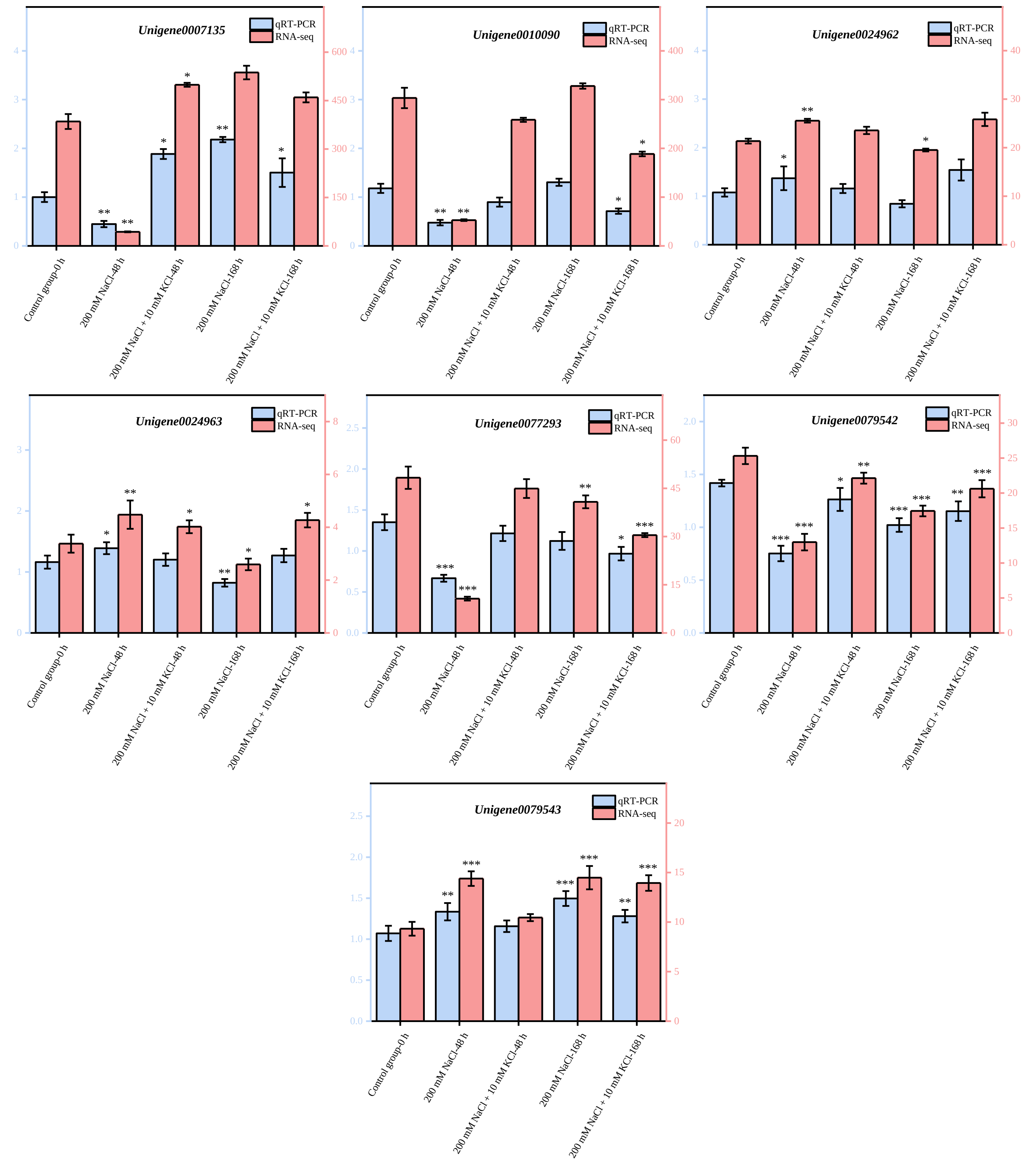

Supplementary Figure S6. Validation of candidate key *WRKY* genes by qRT-PCR

(Seven *WRKY* genes were randomly selected for qRT-PCR validation, and the error bars were obtained from multiple replicates of qRT-PCR. The trends of qRT-PCR value and the RNA-seq value for each gene were consistent and the transcription sequencing results were reliable. Note: $p \geq 0.05$ is not marked; $0.01 < p < 0.05$ is marked as *; $0.001 < p < 0.01$ is marked as **; $p \leq 0.001$ is marked as ***; : Numerical value has been shown on the left side of the *Y* axis; : Numerical value has been shown on the right side of the *Y* axis; qRT-PCR value is the relative quantitative value, and RNA-seq value is the fpkm value).
